# Supplementary material for: The conformational plasticity of structurally unrelated lipid transport proteins correlates with their mode of action
Source: PLoS Biol. 2024 Aug 19;22(8):e3002737. doi: 10.1371/journal.pbio.3002737 (PMC11361750; doi:10.1371/journal.pbio.3002737)
Supplement: S1 Text — Table A. Total variance from the PCA of all the proteins considered in this work along with the contributions from the 2 first principal components. Fig A. Time trace of minimum distance values between the protein and the bilayer, for each replica of simulation; indicates transient and reversible interactions. Fig B. Residue-wise frequency of interaction with the lipid bilayer. Residues that have been experimentally proposed to be crucial for membrane binding are highlighted in blue. Fig C. Interaction frequency of each amino acid with the lipid bilayer, shown for each LTP in our dataset. Fig D. Residue-wise contribution to principal component PC1 for the apo (green), holo (blue), and combined (red) trajectories. GM2A and LCN1 were simulated in the apo-form alone due to the lack of a lipid-bound crystal structure of the protein. Fig E. Comparison of the apo-like (orange) and holo-like (purple) structures of the LTDs arising from the extreme ends of the clustering procedure, and the RMSD between them. Osh4 and Osh6 not shown as the N-terminal lid of the protein that exhibits the largest motion can exist in several folded and unfolded states resulting in a wide range of RMSD values. Fig F. Cavity volumes for apo (orange) and holo (purple) forms of the protein, computed on the projections of PC1. The cavity volumes for the different clusters (C1, C2, and C3) follow the same colour scheme and notations as in Fig 4C. Fig G. Comparison between the overlap of apo-wt (blue)–holo-wt (green) distributions and apo-mutant (red)–holo-mutant (orange) distributions for all mutant proteins considered in this work. The KL divergence values are depicted on the right side. Fig H. PCA of mutants. Residue-wise contribution to principal component PC1 for the wt-apo (green), wt-holo (blue), and mutant-apo (red) for STARD11, GRAMD1A, CPTP, and PITPA. Fig I. Angle between the membrane normal and the 2 principal protein axes (red and orange) in CG-MD simulations of membrane binding for GM2A [file pbio.3002737.s001.docx]

**The conformational plasticity of structurally unrelated lipid transport proteins correlates with their mode of action**

Sriraksha Srinivasan^1&^, Andrea Di Luca^1&^, Daniel Álvarez ^1,2^, Arun T. John Peter^1^, Charlotte Gehin^3^, Museer A. Lone^4^, Thorsten Hornemann^4^, Giovanni D’Angelo^3^ and Stefano Vanni^1,5*^

^1^Department of Biology, University of Fribourg, Fribourg, Switzerland

^2^ Departamento de Química Física y Analítica, Universidad de Oviedo, Oviedo, Spain

^3^Institute of Bioengineering (IBI), and Global Heath Institute (GHI) École Polytechnique Fédérale de Lausanne (EPFL), Lausanne, Switzerland

^4^ Institute of Clinical Chemistry, University Hospital Zurich, University of Zurich, Zurich, Switzerland.

^5^ National Center of Competence in Research Bio-inspired Materials, University of Fribourg, Fribourg, Switzerland

^&^These authors contributed equally

^*^ stefano.vanni@unifr.ch

**Supplementary Information**

|  | **Apo** | | | |  |
| --- | --- | --- | --- | --- | --- |
| **Proteins** | **Total Variance** | **PC1** | **PC2** | **Contrib.**  **PC1 (%)** | **Contrib.**  **PC2 (%)** |
| CPTP | 0.7438 | 0.2775 | 0.1178 | 37.31 | 15.84 |
| ESYT2 | 0.8103 | 0.2948 | 0.1888 | 36.38 | 23.30 |
| FABP | 0.8263 | 0.3089 | 0.1561 | 37.38 | 18.90 |
| GRAMD1A | 0.6515 | 0.1792 | 0.1197 | 27.51 | 18.38 |
| GRAMD1C | 0.6994 | 0.3106 | 0.0845 | 44.41 | 12.08 |
| PITPA | 0.6986 | 0.2131 | 0.1704 | 30.51 | 24.40 |
| OSH4 | 0.7264 | 0.2144 | 0.1383 | 29.52 | 19.03 |
| OSH6 | 0.6575 | 0.1595 | 0.1203 | 24.26 | 18.30 |
| SFH1 | 0.6978 | 0.2782 | 0.1145 | 39.86 | 16.41 |
| TTPA | 0.5991 | 0.2018 | 0.0872 | 33.68 | 14.56 |
| GM2A | 0.8877 | 0.5948 | 0.1271 | 67.00 | 14.32 |
| LCN1 | 0.8356 | 0.4617 | 0.1184 | 55.25 | 14.17 |
|  |  |  | **Average (%):** | 38.59 | 17.47 |

|  | **Holo** | | | |  |
| --- | --- | --- | --- | --- | --- |
| **Proteins** | **Total Variance** | **PC1** | **PC2** | **Contrib.**  **PC1 (%)** | **Contrib.**  **PC2 (%)** |
| CPTP | 0.8003 | 0.3769 | 0.1954 | 47.09 | 24.41 |
| ESYT2 | 0.7024 | 0.2493 | 0.1435 | 35.50 | 20.43 |
| FABP | 0.8953 | 0.5283 | 0.1248 | 59.01 | 13.94 |
| GRAMD1A | 0.6991 | 0.2143 | 0.1388 | 30.65 | 19.86 |
| GRAMD1C | 0.6105 | 0.1671 | 0.1139 | 27.38 | 18.65 |
| PITPA | 0.6431 | 0.1892 | 0.1239 | 29.42 | 19.27 |
| OSH4 | 0.7075 | 0.2161 | 0.1430 | 30.54 | 20.21 |
| OSH6 | 0.6164 | 0.1441 | 0.1065 | 23.38 | 17.28 |
| SFH1 | 0.4872 | 0.1236 | 0.0743 | 25.38 | 15.25 |
| TTPA | 0.5528 | 0.1506 | 0.1029 | 27.25 | 18.61 |
|  |  |  | **Average (%):** | 33.56 | 18.79 |

|  | **Apo+Holo** | | | |  |
| --- | --- | --- | --- | --- | --- |
| **Proteins** | **Total Variance** | **PC1** | **PC2** | **Contrib.**  **PC1 (%)** | **Contrib.**  **PC2 (%)** |
| CPTP | 0.7783 | 0.2530 | 0.2053 | 32.50 | 26.38 |
| ESYT2 | 0.7940 | 0.3869 | 0.1723 | 48.72 | 21.71 |
| FABP | 0.8512 | 0.3734 | 0.1347 | 43.87 | 15.83 |
| GRAMD1A | 0.6550 | 0.1744 | 0.1413 | 26.62 | 21.57 |
| GRAMD1C | 0.6354 | 0.2264 | 0.1005 | 35.63 | 15.82 |
| PITPA | 0.6408 | 0.1659 | 0.1172 | 25.89 | 18.29 |
| OSH4 | 0.6894 | 0.2113 | 0.1117 | 30.65 | 16.20 |
| OSH6 | 0.6271 | 0.1941 | 0.0887 | 30.95 | 14.14 |
| SFH1 | 0.7983 | 0.5155 | 0.0918 | 64.57 | 11.50 |
| TTPA | 0.6381 | 0.2696 | 0.0781 | 42.25 | 12.24 |
|  |  |  | **Average (%):** | 38.17 | 17.37 |

**Table A.** Total variance from the PCA of all the proteins considered in this work along with the contributions from the two first principal components.


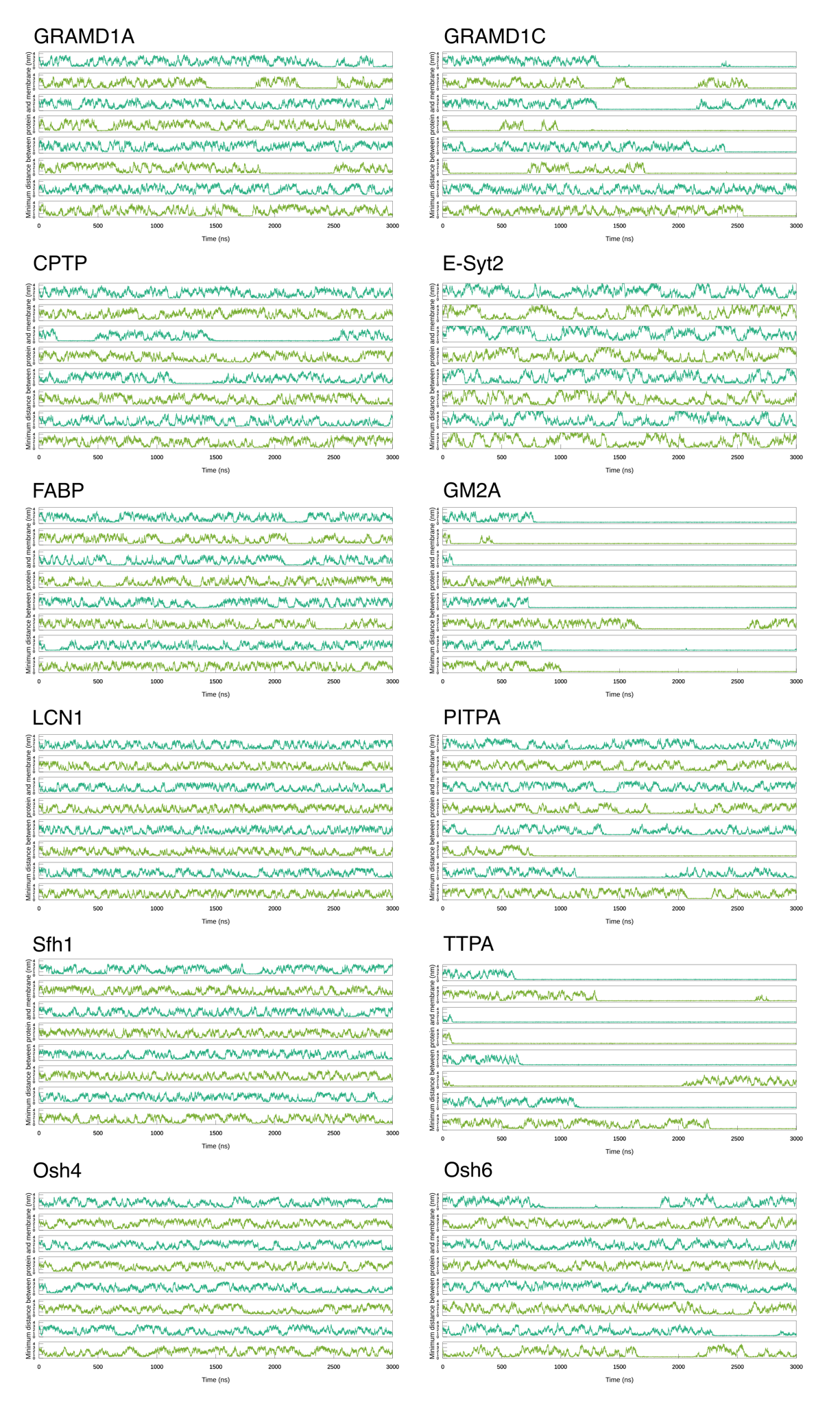


**Fig A.** Time trace of minimum distance values between the protein and the bilayer, for each replica of simulation; indicates transient and reversible interactions. The data underlying the graphs shown in the figures can be found in <https://doi.org/10.5281/zenodo.12728271>


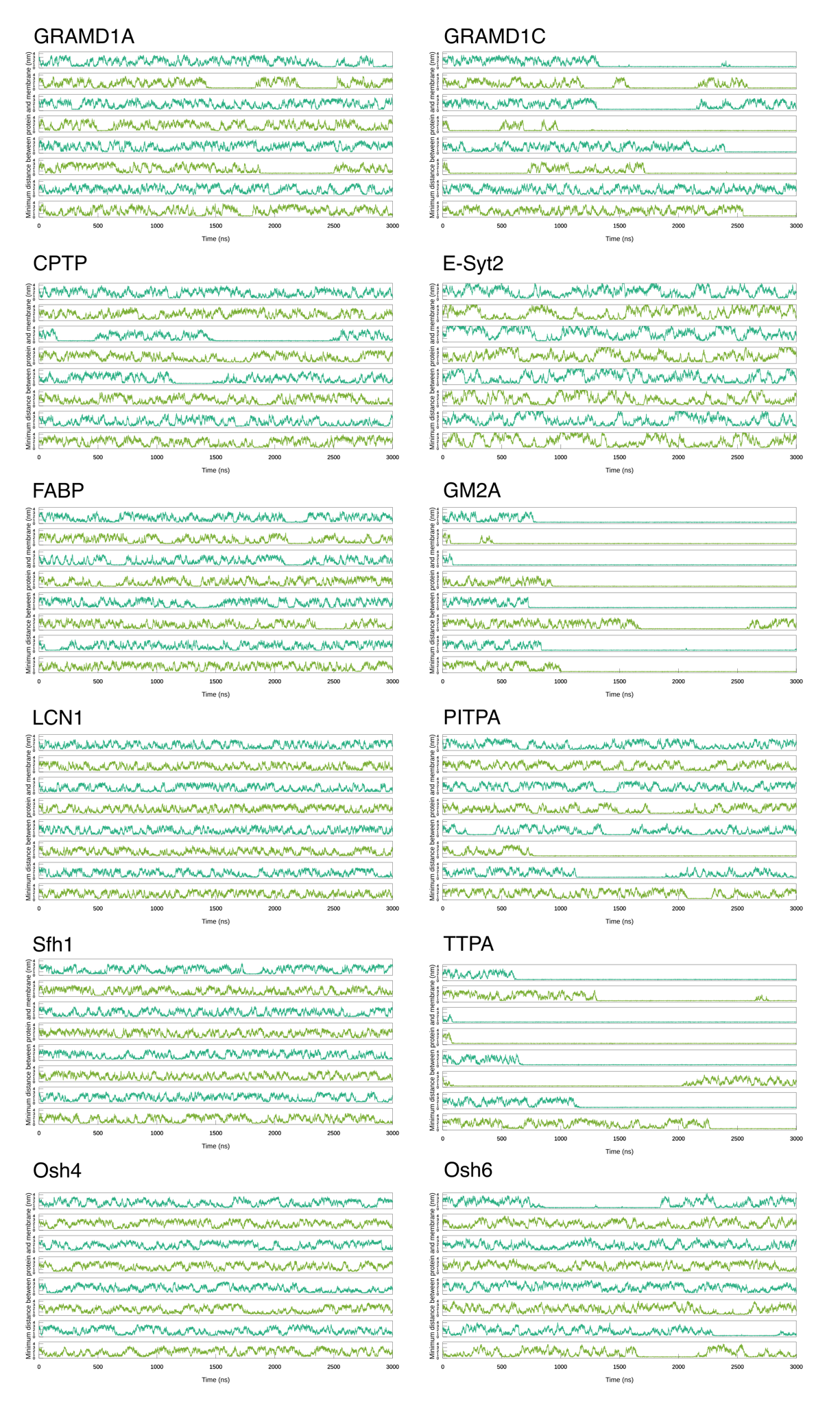


**Fig A (cont.).** Time trace of minimum distance values between the protein and the bilayer, for each replica of simulation; indicates transient and reversible interactions. The data underlying the graphs shown in the figures can be found in <https://doi.org/10.5281/zenodo.12728271>


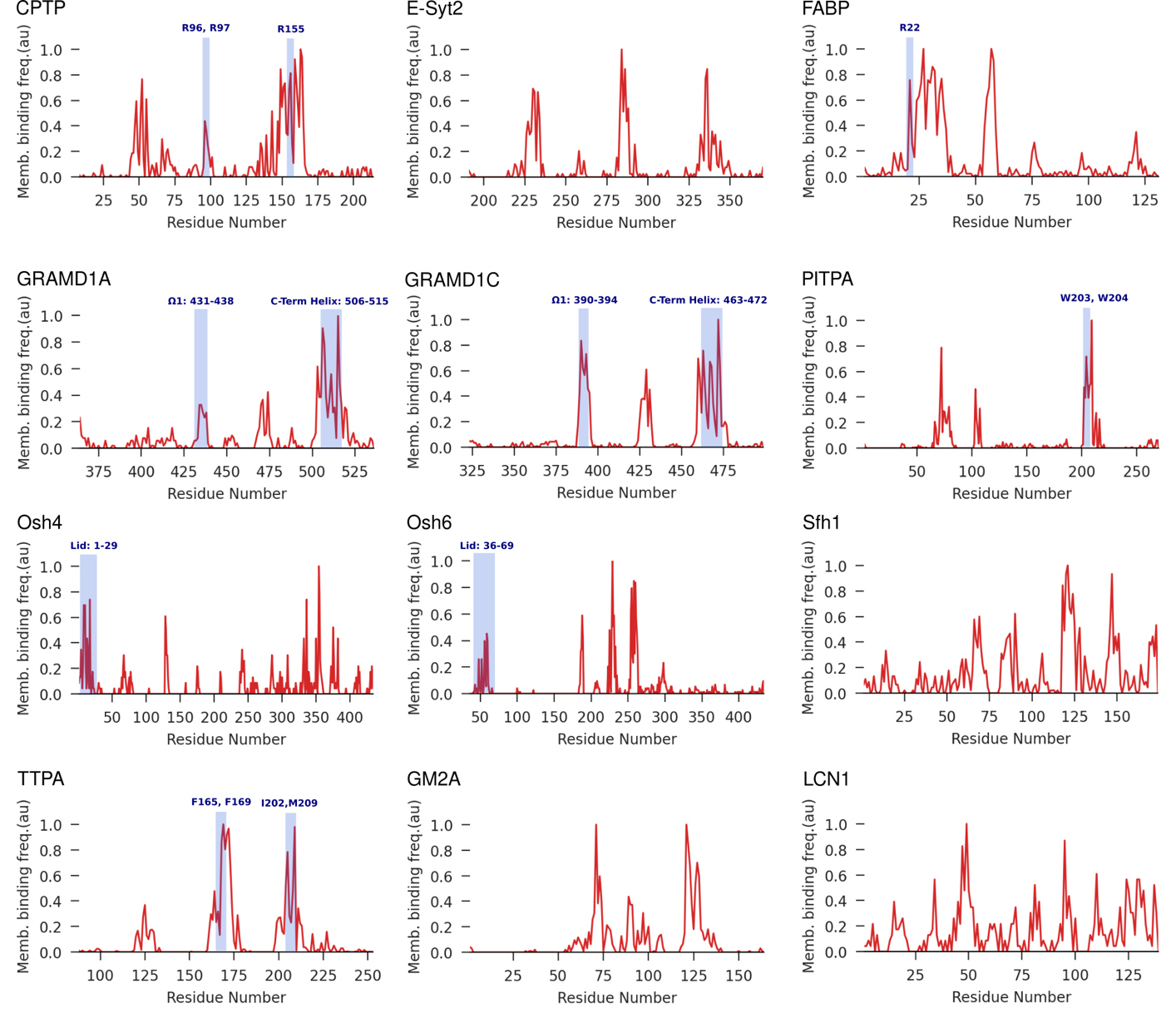


**Fig B.** Residue-wise frequency of interaction with the lipid bilayer. Residues that have been experimentally proposed to be crucial for membrane binding are highlighted in blue. The data underlying the graphs shown in the figures can be found in <https://doi.org/10.5281/zenodo.12728271>


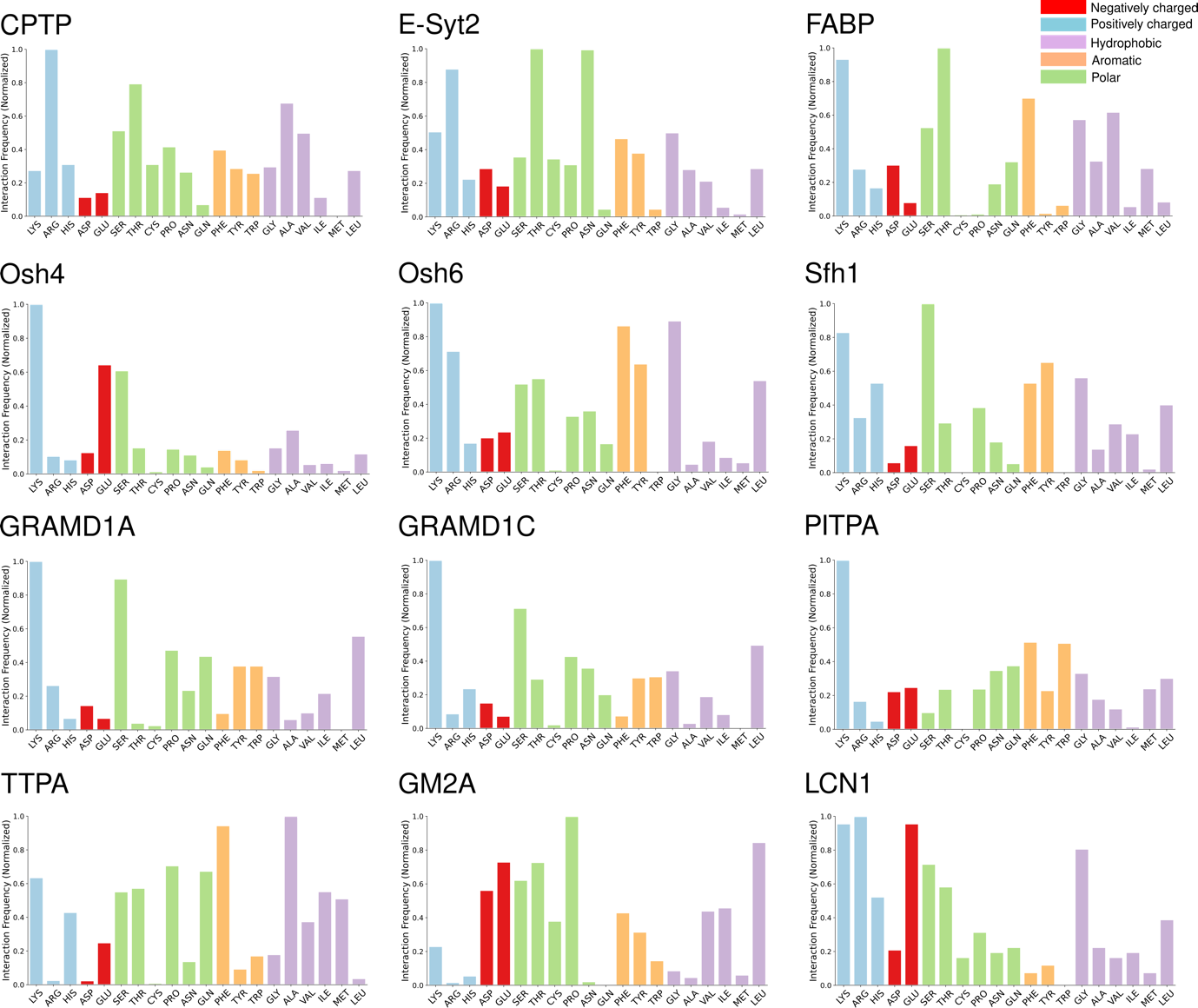


**Fig C**. Interaction frequency of each amino acid with the lipid bilayer, shown for each LTP in our dataset. The data underlying the graphs shown in the figures can be found in <https://doi.org/10.5281/zenodo.12728271>


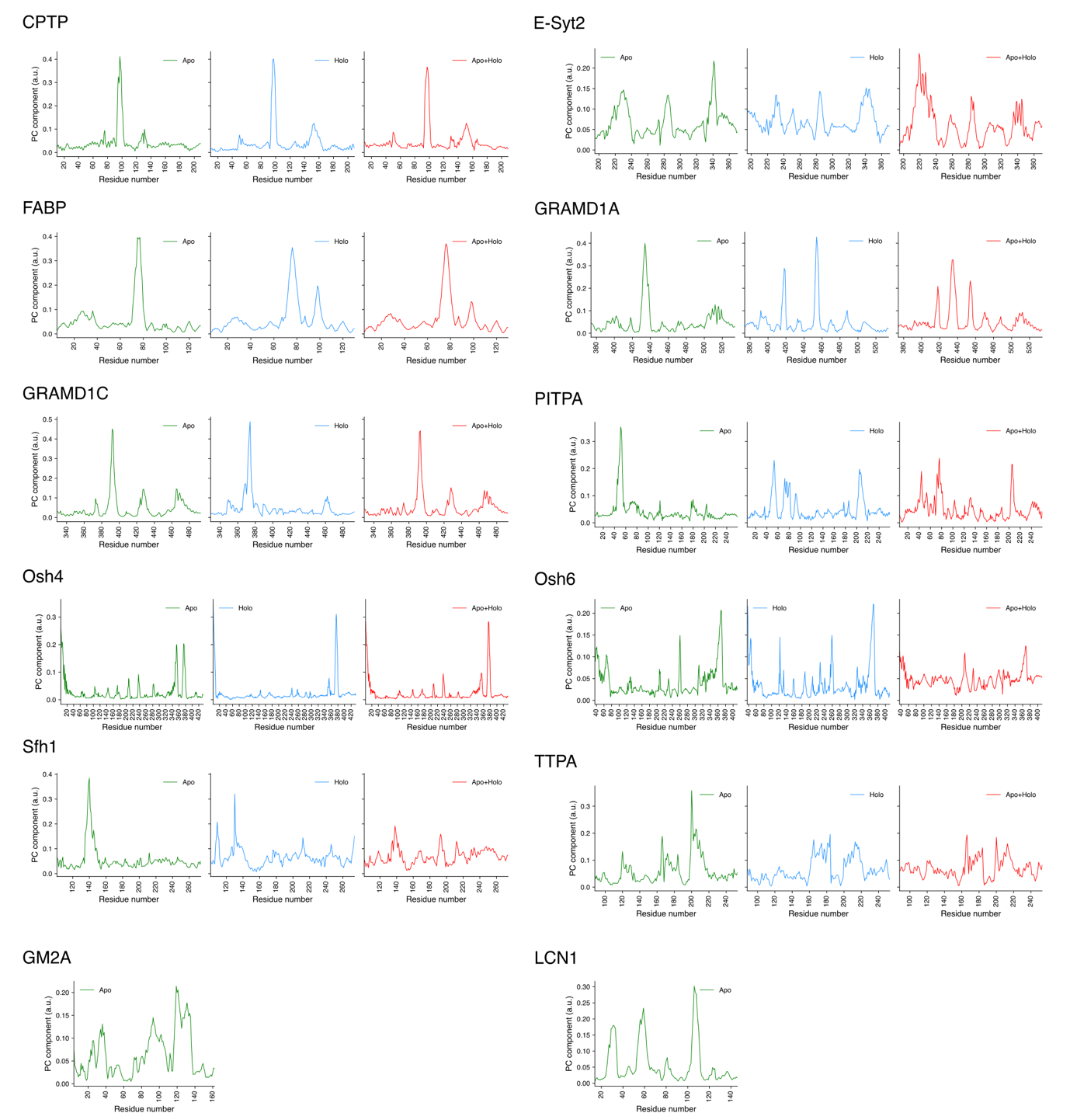


**Fig D.** Residue-wise contribution to principal component PC1 for the apo (green), holo (blue), and combined (red) trajectories. GM2A and LCN1 were simulated in the apo-form alone due to the lack of a lipid-bound crystal structure of the protein. The data underlying the graphs shown in the figures can be found in <https://doi.org/10.5281/zenodo.12728271>


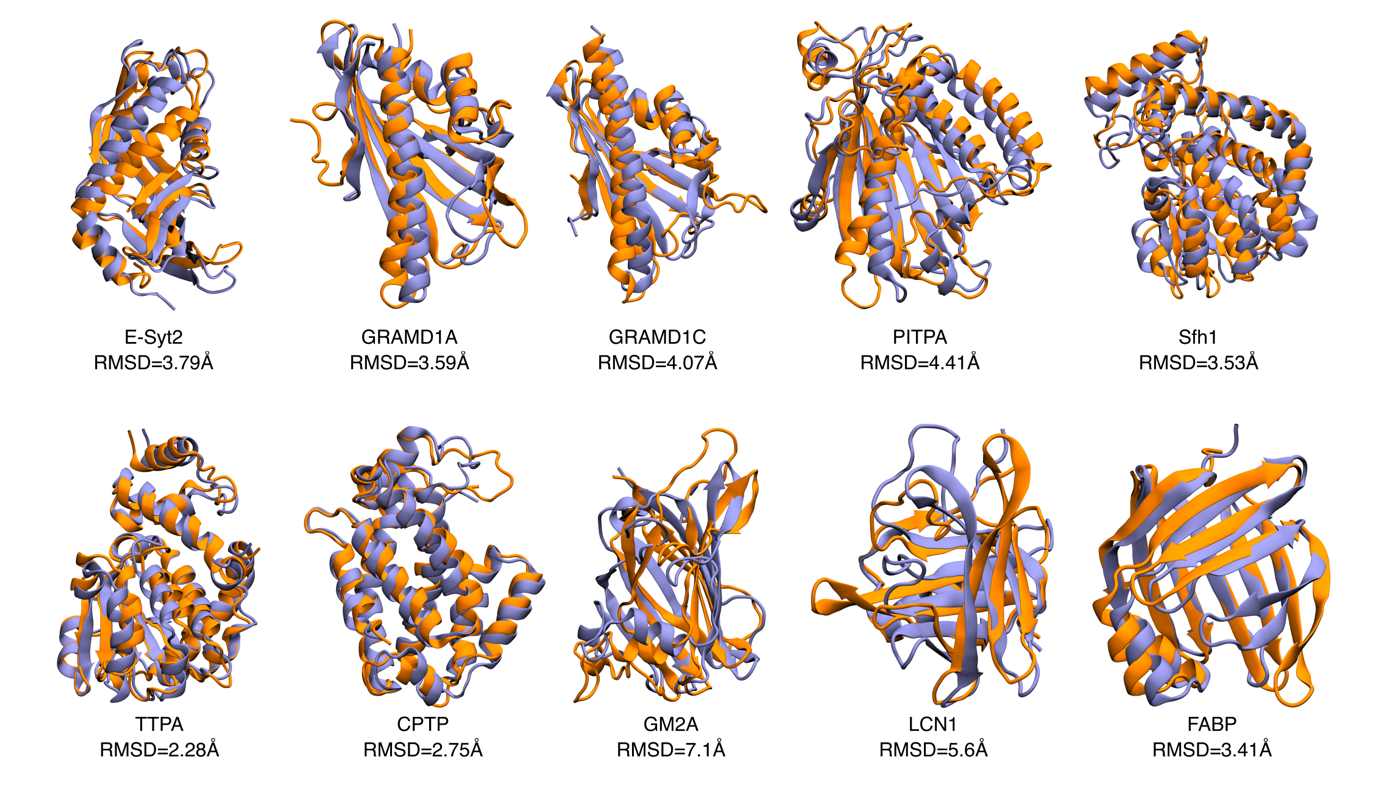


**Fig E.** Comparison of the apo-like (orange) and holo-like (purple) structures of the LTDs arising from the extreme ends of the clustering procedure, and the RMSD between them. Osh4 and Osh6 not shown as the N-terminal lid of the protein that exhibits the largest motion can exist in several folded and unfolded states resulting in a wide range of RMSD values. The data underlying the graphs shown in the figures can be found in <https://doi.org/10.5281/zenodo.12728271>


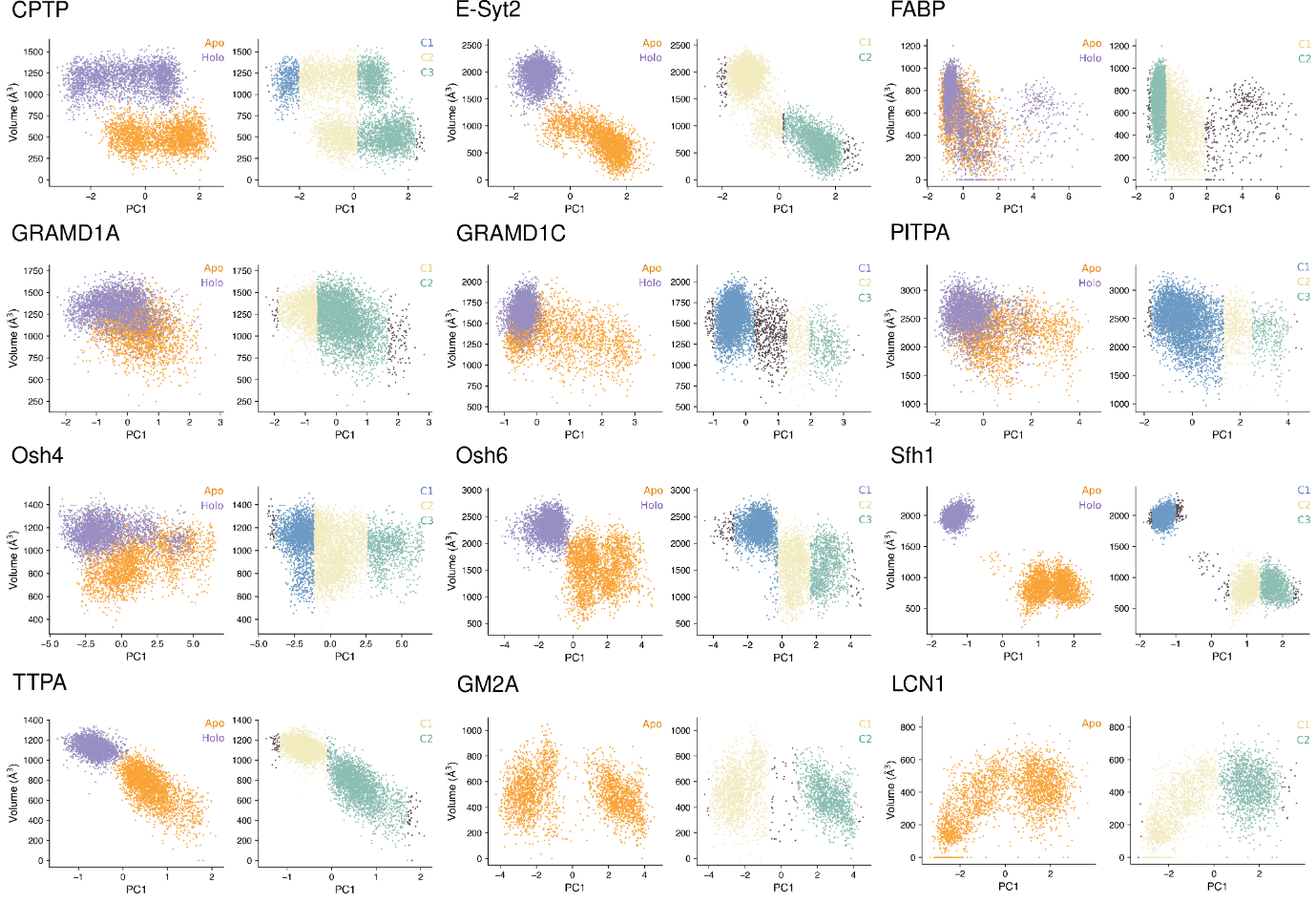


**Fig F.** Cavity volumes for apo (orange) and holo (purple) forms of the protein, computed on the projections of PC1. The cavity volumes for the different clusters (C1, C2, C3) follow the same colour scheme and notations as in Figure 4c. The data underlying the graphs shown in the figures can be found in <https://doi.org/10.5281/zenodo.12728271>


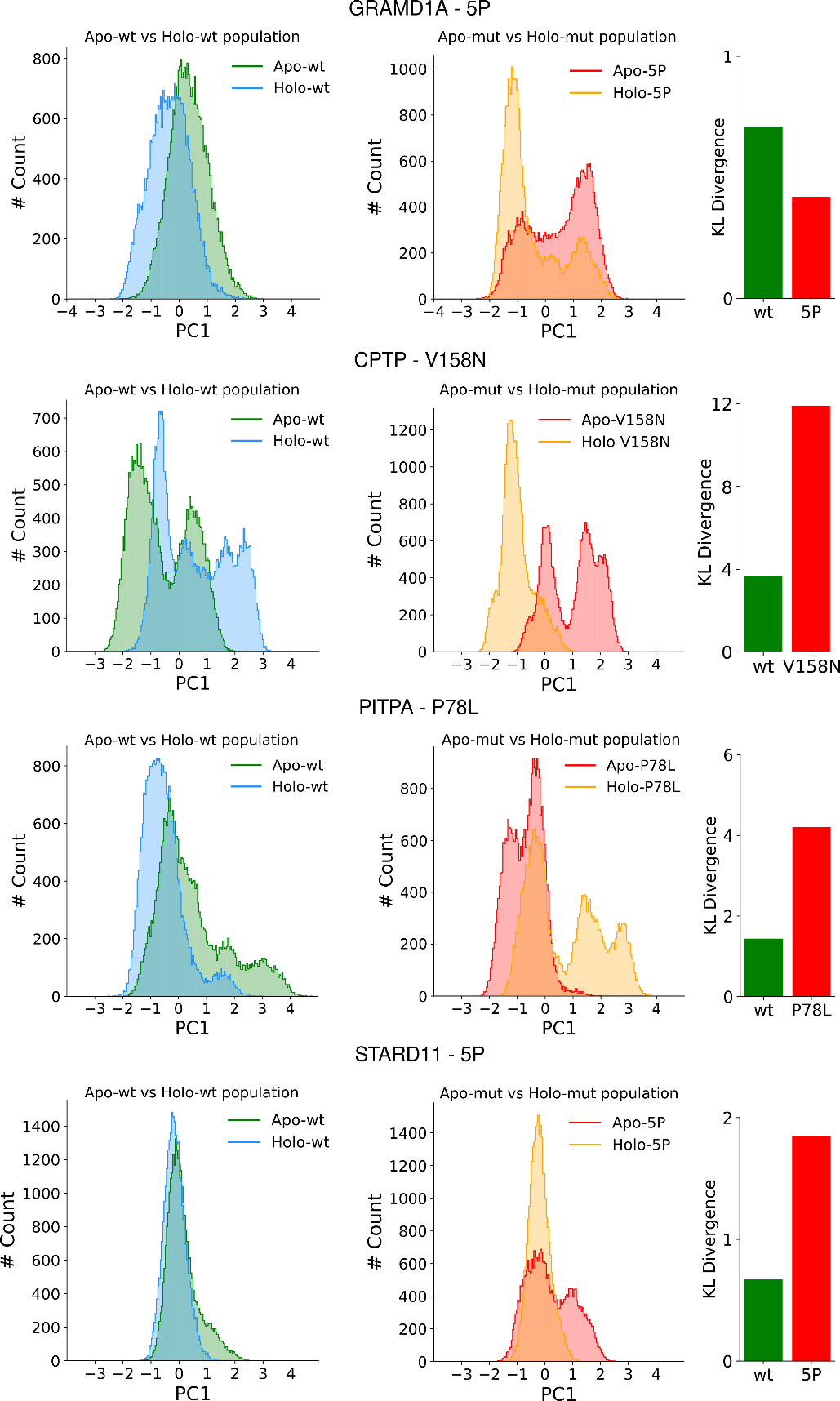


**Fig G.** Comparison between the overlap of apo-wt (blue) – holo-wt (green) distributions and apo-mutant (red) – holo-mutant (orange) distributions for all mutant proteins considered in this work. The KL divergence values are depicted on the right side. The data underlying the graphs shown in the figures can be found in <https://doi.org/10.5281/zenodo.12728271>


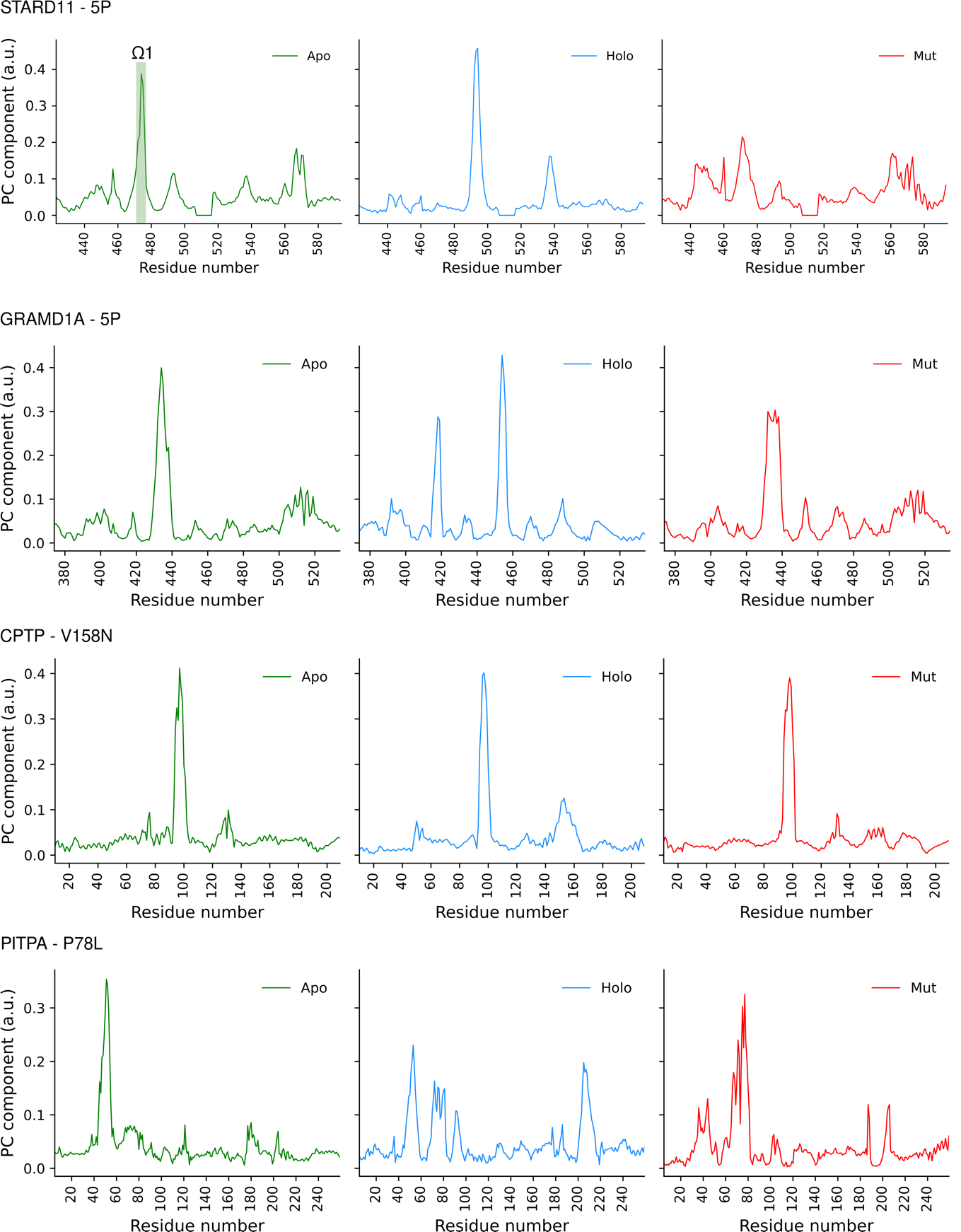


**Fig H.** PCA of Mutants. Residue-wise contribution to principal component PC1 for the wt-apo (green), wt-holo (blue), and mutant-apo (red) for STARD11, GRAMD1A, CPTP, and PITPA. The data underlying the graphs shown in the figures can be found in <https://doi.org/10.5281/zenodo.12728271>


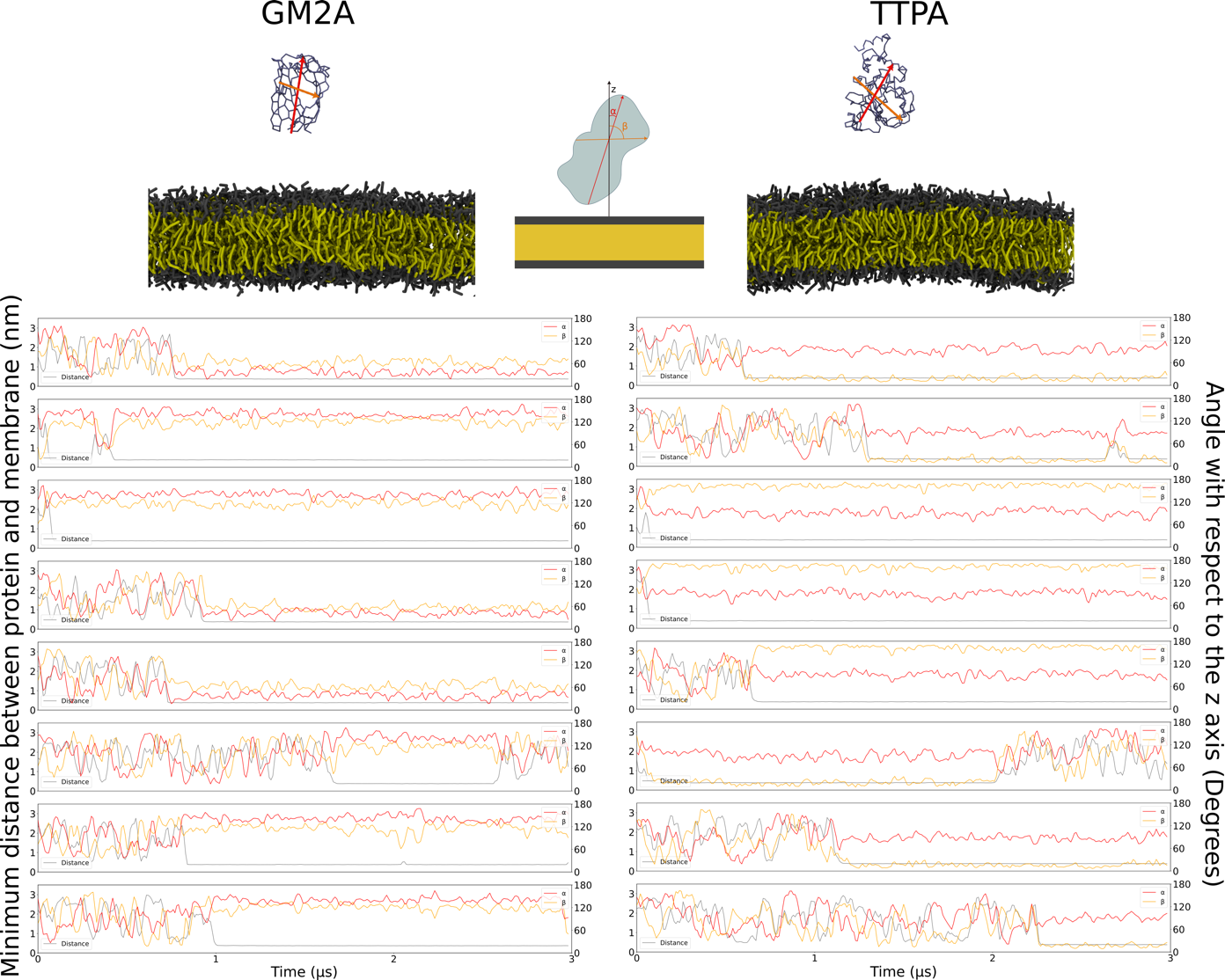


**Fig I.** Angle between the membrane normal and the two principal protein axes (red and orange) in CG-MD simulations of membrane binding for GM2A (left) and TTPA (right) along with the minimum distance values between the proteins and the bilayer (gray) for all replicas All values have been smoothed by doing block average every 2 ns. The data underlying the graphs shown in the figures can be found in <https://doi.org/10.5281/zenodo.12728271>

**
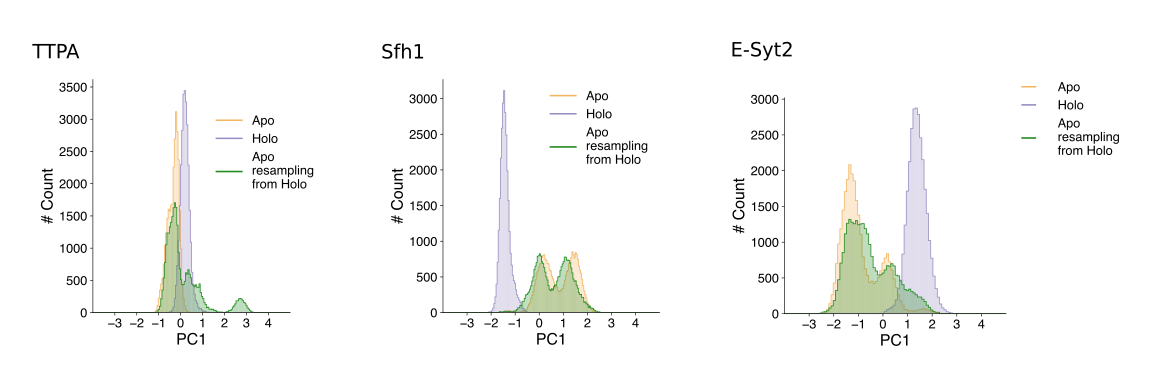
**

**Fig J.** The population distributions of PC1 from simulations of apo form (orange), holo form (purple), and simulations of the protein in the lipidless-form starting from the final structure obtained from the holo simulations by removing the lipid from the last frame of the simulations (green). The PC obtained from resampling (green) are similar to the ones obtained from simulating the apo form of the LTD starting from the crystal structure (orange), indicating that all conformational changes observed are reversible and the protein is not trapped in a metastable state. The data underlying the graphs shown in the figures can be found in <https://doi.org/10.5281/zenodo.12728271>
