# Supplementary figures and images for: The conformational plasticity of structurally unrelated lipid transport proteins correlates with their mode of action
Source: PLoS Biol. 2024 Aug 19;22(8):e3002737. doi: 10.1371/journal.pbio.3002737 (PMC11361750; doi:10.1371/journal.pbio.3002737)

anti-CERT 60 sec exposure

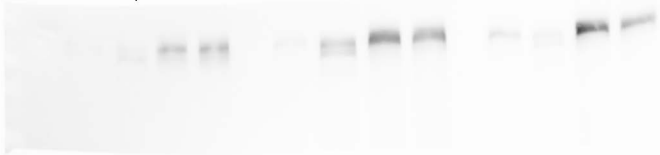

anti-CERT 120 sec exposure

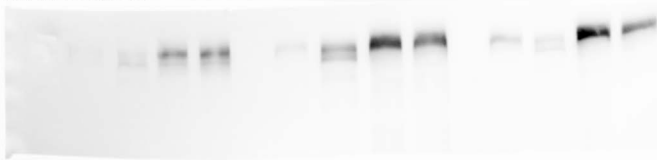

anti-GAPDH 10 sec exposure

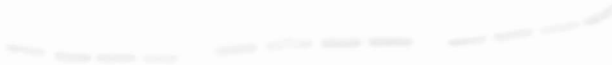

Supplement: S1 Raw Images — (PDF) [file pbio.3002737.s002.pdf]
