# Supplementary material for: The conformational plasticity of structurally unrelated lipid transport proteins correlates with their mode of action
Source: PLoS Biol. 2024 Aug 19;22(8):e3002737. doi: 10.1371/journal.pbio.3002737 (PMC11361750; doi:10.1371/journal.pbio.3002737)
Supplement: Attachment — Submitted filename: 135821_1_rebuttal_2723864_rx809r.pdf [file pbio.3002737.s003.pdf]

# Revision Plan

**Manuscript number:** RC-2023-.1961
**Corresponding author(s):** Stefano, Vanni

*[The "revision plan" should delineate the revisions that authors intend to carry out in response to the points raised by the referees. It also provides the authors with the opportunity to explain their view of the paper and of the referee reports.*

*The document is important for the editors of affiliate journals when they make a first decision on the transferred manuscript. It will also be useful to readers of the reprint and help them to obtain a balanced view of the paper.*

## 1. General Statements [optional]

***We thank the reviewers for acknowledging the relevance and impact of our findings in the field of lipid transport and metabolism. In this "revision plan", we have (i) addressed all concerns by Reviewer #1 and #3, and (ii) discussed our plan to address the main concerns of Reviewer #2. Overall, while we acknowledge that a final proof of our hypothesis would require extensive in vitro validation, this is beyond the scope of the current study (and beyond our current technical capabilities). We are of the opinion, however, that additional analyses and simulations suggested by Reviewer #2, together with careful rewriting of some parts of the manuscripts, to avoid mis- or over-interpretation, would result in a contribution that significantly advances our current knowledge on structure-dynamics-function of lipid transport by LTPs.***

## 2. Description of the planned revisions

*Insert here a point-by-point reply that explains what revisions, additional experimentations and analyses are <u>planned</u> to address the points raised by the referees.*

**Reviewer #2** *(Evidence, reproducibility and clarity (Required)):*

*Summary:*

*In a combined computational and experimental study, the authors provide insights into general features of lipid transfer proteins (LTPs), which play key roles in lipid trafficking: Through molecular dynamics simulations of a diverse set of 12 shuttle-like LTPs, they demonstrate that LTPs consistently exist in an equilibrium between two or more conformations, whose populations are modulated by a bound lipid, and that residues significantly involved in these collective conformational changes typically interact with a membrane. Their simulations indicate that conformational plasticity is a general feature of LTPs, leading them to suggest that the ability to change conformations is essential for LTP function. They test the generality of this hypothesis through in cellulo assays of two LTPs (STARD11 and Mdm12) that were not*

*originally simulated. While experiments of STARD11 support their hypothesis, those presented for Mdm12 provide ambiguous results.*

*Major comments:*

*Throughout the manuscript, it's stated that common 'dynamical features' correlate with LTP function. The accuracy of this statement is unclear since 'dynamical features' are never precisely defined and, while equilibrium conformational ensembles are characterized, dynamics (ie kinetics or time-dependent observables) are not. Please clarify.*

***We plan to improve the scholarly presentation of our article to clarify this issue. In short, two distinct properties modulate protein function: 1. Conformational plasticity, i.e. the (thermodynamic) ability of the protein to adopt different conformations (and with different populations depending on the bound substrate). 2. Conformational "dynamics", i.e. the propensity to exchange between these different thermodynamic states. This ability depends on the free energy barriers between different states and it is intrinsically a kinetic (rather than thermodynamic) property.***

*More importantly, further evidence is needed to determine a correlation with *function*. LTPs are suggested to have faster transfer rates (a measure of function) if the apo form adopts a substantial population of holo-like conformations, akin to enzyme preorganization. This is further tested by rationally mutating STARD11 and Mdm12. However, the support for this conclusion and if these mutations alter the LTPs conformational ensembles as desired is unclear:*

***In our opinion, the interpretation suggested by Reviewer #2 that there is a "correlation" between transfer rates and the overlap of apo-like and holo-like conformations, though fascinating, cannot be derived from the available data at this stage, and we did not mean to imply as such. Rather, lipid transport is a complex phenomenon that involves several steps (membrane binding/unbinding, lipid uptake/release,…). Our simulations indicate that protein conformational plasticity, including potentially the overlap between apo-like and holo-like conformations, also influences lipid transfer rates. We will clarify this aspect in the text.***

*• Is there a quantitative correlation between the overlap of apo and holo conformational distributions (as could be quantified by KL divergence or Wasserstein distance, for example) and difference in transfer rates as suggested by Fig S6?*

***We plan to compute quantitative correlation between apo and holo conformational distribution for Fig.S6 and for mutant simulations (see answer below) but, as discussed above, we are skeptical that we will observe a clear correlation.***

*• The conclusion and the generality of the findings would be greatly strengthened if a correlation*

*can be shown for other LTPs through additional simulations of mutants whose transfer rates have been previously characterized experimentally in the literature. (For example: Ryan 2007 PMID 17344474, Grabon 2017 PMID 28718450, Iaea 2015 PMID 26168008, among many others)*

***We are currently running simulations of several mutants to address this point and provide additional data/context.***

*• While differences in the apo conformational ensembles of the WT and mutants are observed in Fig S7b and d, if these mutations reduce overlap with holo-like conformations is not determined. Simulations of the WT holo forms are needed to properly test this hypothesis.*

***We are currently performing these simulations.***

*• For Mdm12, mutations are specifically made to "lock the protein in the apo-like state;" however, the mutant adopts conformations distinct from the apo form as show in Fig S7d. How do the authors interpret the results of the cellular assays considering this and could it help explain why the mutant has similar kinetics to WT? What may explain the puzzling results of similar transfer kinetics but differing mitochondrial morphology?*

***As discussed above, interpretation of lipid transport rates based exclusively on apo and holo conformational population is premature, as this is a complex mechanism that depends on many variables. For what concerns the experimental results, we think three explanations are possible: 1. Mitochondrial morphology could be more sensitive to small variations in lipid composition than our METALIC assay. 2. Our assay only quantifies transport of unsaturated PC and PE species, and we can't quantify variations in transport of other lipid species that are likely to also be transported by ERMES, such as PS and PA. 3. According to a recent structural model (Wozny et al, Nature 618, 88–192, 2023), Mdm12 might be part of a tunnel-like LTP complex in which it doesn't establish direct interactions with nearby organellar membranes. As such, its mechanism might be different from the one described here for other shuttle-like lipid transport domains. We will discuss these possibilities in the main text.***

*• The abstract, intro, and title highlight that the manuscript's findings are indicative of and correlated with *function* but on p. 12 it's foreseen "that future studies will focus on the functional consequence of such observation." Please reconcile these conflicting statements and ensure connections to function are accurately described. The current title is rather bold.*

***We will rewrite and clarify the extent of our hypotheses and validations.***

*All mentions of "correlation" throughout the manuscript need to be quantitatively evaluated or properly qualified. In addition to that mentioned above regarding Fig S6, what is the correlation coefficient between residues' contribution to PC1 and membrane interaction frequency (Fig 2)?*

***To address this point, we will quantify the correlation between residues' contribution to PC1 and membrane interaction frequency. However, we expect a low correlation between residues' contribution to PC1 and membrane interaction frequency for at least two main reasons. First, not all residues contributing to PC1 interact with membranes, but only a subset, as discussed above. Second, our methodology to compute membrane binding, based on the geometric distance between residues and bilayer, is intrinsically quite noisy (since residues in proximity of bona fide membrane binding regions will also appear as involved in membrane binding), thus making quantification of correlations somewhat inaccurate. Rather, we will try to explain in the text that our observations are not of "correlation" but rather of dependence/association, and we will use quantitative measures to quantify these properties (such as rank correlation coefficients or multivariate analyses).***

*Residue's contributions to collective conformational changes are found to be indicative of membrane binding. Yet, membrane interacting residues are identified from CG simulations that cannot capture such collective conformational changes due to the use of an elastic network. Given that the CG simulations agree with previous experimental findings, this suggests that collective conformational changes are not important for membrane binding.*

***We disagree with this interpretation by Reviewer #2 of our data: we do not claim that residue's contributions to collective conformational changes is indicative of membrane binding. Rather, membrane binding happens at protein regions displaying high contribution to collective conformational changes. This distinction is subtle but important: protein motion does not determine membrane binding regions. Rather, it appears that, for LTPs, membrane binding regions are also characterized by collective motions (suggesting function). We will clarify this in the main text.***

*Are similar conclusions drawn from residues' RMSFs? In other words, are local conformational fluctuations just as indicative of membrane binding?*

***We will compute protein residues' RMSFs and compare it with the membrane binding data. However, given that RMSF is representative of thermal fluctuations, we again expect a bad correlation between RMSF and membrane binding. On the other hand, we indeed observe that most membrane binding regions are protein loops, but this is not unexpected (e.g. Tubiana et al, PLoS Comput Biol. 2022 Dec; 18(12): e1010346.). However, such observation does not provide any information on lipid transport, but only on the mechanism of membrane binding. Rather, the observation of a relationship between membrane binding and global motion is more interesting, since the latter is often indicative of protein function.***

*The stated correlation may in fact be spurious and instead arise because residues at the entrance to LTP's hydrophobic cavities need to be positioned at the membrane surface for productive lipid uptake and these same residues must undergo significant conformational changes to allow lipid entry.*

***This is exactly what we think it is happening and what our data suggest. However, one must remember that our simulations allow us to predict the membrane binding interface, that is often difficult to determine experimentally (and often via indirect evidence). Hence our data provide novel evidence in this direction.***

*Is proximity to cavity entrance more or less correlated with membrane binding than 'dynamics'?*

***If we consider that, as discussed before, dynamics does not correlate with membrane binding (there are many dynamical regions that are not at the membrane interface), it is safe to assume that proximity to cavity entrance would correlate more with membrane binding. However, we have to consider that often we do not know where the cavity entrance in LTPs is located simply based on structure alone, and hence our approach provides important clues into this process.***

*p. 12 speculatively suggests "the high degree of protein dynamics we observed in membrane proximal regions could potentially facilitate the energetically unfavorable reaction that involves the extraction of a lipid from a membrane." Yet, the logic behind this idea does not make sense since a free energy barrier, an equilibrium thermodynamic quantity, cannot be lowered by changes in dynamics. Please explain.*

***Our current understanding of the mechanism of lipid extraction is quite poor. However, both using chemical intuition and following a recent MD study on one LTP (Rogers et al, 2023, Plos Comp Biol), it is safe to assume that the hydrophobic environment around the lipid is important for its stabilization in the lipid bilayer. Hence, reducing the number of hydrophobic contacts between the lipid and its environment could facilitate transport. A highly dynamic protein, by cycling between different conformations, could "stir" the bilayer, and hence decrease the number of contacts between the lipid and its environment favoring transport. We will clarify this point in the text.***

*Examining how the LTPs impact membrane properties would offer insight into the functional relevance of such residues for lipid extraction.*

***Indeed, our point above is connected to this one. We are performing simulations to compute hydrophobic contacts in bilayer as proposed in (Rogers et al, 2023, Plos Comp Biol).***

*The authors motivate the study with the *assumption* that a common molecular mechanism of*

*LTP function exists. Yet LTPs have evolved diverse sequences, structures, and substrate preferences; thus there seems to be no a priori requirement (or even necessarily a benefit) for a single molecular mechanism. What evidence then supports this premise? While previous studies are limited to individual LTPs, when viewed altogether retrospectively, they suggest features that could be shared among LTPs. Synthesizing previous studies and more thoroughly referencing them (only 5 are cited in the intro on p. 3) would strengthen both the premise and findings of the manuscript.*

***Indeed, despite having different structures, substrates and the ability to target distinct organelles, previous evidence on LTPs seem to suggest a potential role for protein conformational plasticity for function, e.g. for Osh/ORP (Jun Im et al, Nature 2005; Canagarajah et al, JMB 2008; Moser von Filseck et al, Nat Comm, 2015; Lipp et al, Nat Comm. 2019,...), StART (Arakane et al, PNAS, 1996; Feng et al, Biochemistry, 2000; Grabon et al, JBC, 2017; Khelashvili et al, eLife, 2019;...) and PITP domains (Tremblay et al, Archives of Biochemistry and Biophysics, 2005; Ryan et al, MBOC, 2007; …). Our simulations provide additional evidence in this direction and allow for generalizing these observations, allowing to draw parallelisms with "enzyme-like" or transporter-like" features that could be exploited for further design of testable hypotheses. We will rewrite our text to better contextualize/acknowledge previous findings and to clarify these points.***

*The LTPs investigated are known to target distinct membranes. Should they then be expected to share structural or sequence-based features predictive of membrane binding interfaces, as motivates the analysis in Fig 1d, 1e, and S3? Or is it beneficial for LTPs to recognize membranes in different ways?*

***Since membrane binding is membrane/organelle-specific, it is possible that residue's diversity in membrane binding interfaces could indeed be beneficial for this diversity. We will add this comment as a potential explanation of our finding of a lack of conserved sequence-based features for membrane binding interfaces.***

*Minor comments:*

*p. 2 "making lipid transfer across the cytoplasm a potentially energetically favorable process": Is it meant that it is less energetically costly than transfer without a LTP? Why it would be energetically favorable is unclear (and would indicate that the LTP sequesters lipids away from membranes instead of transferring them between membranes).*

***Yes, this is what we meant. We will rewrite this appropriately.***

*p. 3 "The excellent agreement between the membrane interface determined from the simulations and the experimentally-proposed one available for... Osh6" is missing a citation.*

***We have now added the relevant citation.***

*The plots in Fig 1d and S3 are difficult to interpret. Bar plots, for example, would allow easier comparison and evaluation. Currently, it seems that most proteins individually exhibit some of the same trends observed among the whole set, counter to the conclusion on p 5.*

***We will improve the presentation of our Figures.***

*Negatively charged residues engage in a number of membrane interactions (Fig 1d and S3). What is a potential explanation for this unconventional observation?*

***One possible interpretation is that negatively charged residues could interact with positively charged moieties (ethanolamine, choline) of PC and PE lipids.***

*How much variance is captured by PC1, and how many PCs are needed to capture most of the variance in the conformations?*

***PC1 explains 38 % of the total variance, by average, whereas PC2 accounts for 17 % of it. Therefore, PC1 and PC2 capture most of the variance in almost all cases.***
***We have also added this to the text:***

***"We specifically focused on PC1 as it explains most of the variance in the dynamics (38% on average for all the proteins in our dataset, see Supplementary Table 2). "***

***We have computed this variance and we have added this analysis in Supplementary Information.***

*Plots in Fig 3, especially panels c and d are difficult to see. Please make the panels larger (perhaps a 3 x 4 layout instead of 2 x 6 would work better).*

***We will improve the presentation of our Figures.***

*p. 8 "these conformational changes are localized in protein regions that interact with the lipid bilayer" is contradicted by the results in Fig 2b showing that all residues with large contributions to PC1 do not interact with the membrane and discussed on p 5.*

***As discussed above, we don't observe "correlation" between membrane binding and conformational plasticity, but we rather observe that membrane binding regions display high conformational plasticity (the opposite is not true). We will further clarify in the text.***

*p. 8 "in the absence of bound lipids, it is able to sample multiple conformations" is not supported*

*by the orange distributions in Fig 3d that appear unimodal. Is it instead meant that the apo form exhibits larger variance in cavity volume?*

***Yes, this is what we meant. We'll clarify.***

*Please clarify if the elastic network was constructed to maintain the holo or apo structures of each protein and if a bound lipid was used in the CG simulations.*

***For membrane binding CG simulations, we used the apo structure and no bound lipid was used in the simulations. However, analogous simulations in the holo form (not shown) have essentially identical membrane binding interfaces.***

*Was *CHARMM* TIP3P used?*

***Yes.***

*Please clarify how membrane interacting residues were defined and how interaction frequency was calculated from the longest duration of interaction.*

***We will add this explanation in the Methods. The method is identical to (Srinivasan et al, Faraday Discussion, 2021)***.

*Refs 16 and 45 refer to the same paper.*

***Thanks, it is now corrected!***

***Reviewer #2*** *(Significance (Required)):*

*General assessment:*

*The work aims to tackle a grand question regarding membrane homeostasis mechanisms-what are universal principles underlying LTP function-and offers initial insights; however, further evidence is needed to support the conclusions as written, and some key results require further investigation and explanation.*

*Advance and audience:*

*By concurrently investigating the largest number of lipid transfer proteins to-date, the authors provide data invaluable for uncovering general mechanisms of non-vesicular lipid transport and advancing our understanding of membrane homeostasis mechanisms. By illuminating the wide-*

*spread importance of conformational plasticity among lipid transfer proteins, the work presents a conceptual advance in our understanding of lipid transfer mechanisms and unifies previous studies. Because the manuscript emphasizes common biophysical principles and draws connections to enzyme biophysics, it ought to be of interest not only to membrane biologists but biochemists and molecular biologists more broadly.*

***We thank Reviewer #2 for the very positive evaluation of the significance of our work and for the in-depth analysis provided that will certainly help improve the quality of our work.***

## 3. Description of the revisions that have already been incorporated in the transferred manuscript

***We report here a point-by-point response to Reviewer #1 and Reviewer #3. We have already addressed all their concerns in the updated version of the manuscript.***

***Reviewer #1** (Evidence, reproducibility and clarity (Required)):*

*Srinivasan et al. present a comprehensive study on systematizing the structure-dynamics-function relation of lipid transfer proteins (LTPs), combining extensive molecular simulations and complementary experiments. Indeed, the current state-of-the-art in the field is quite chaotic and fractional, and such systematic studies are necessary to advance our general and conceptual understanding of the mechanisms of action of LTPs. The selected techniques and research strategies are all suitable, their description is sufficient and enables reproducibility; the obtained results are carefully presented and discussed; the conclusions are adequately supported by the data.*

*Given my primarily computational background, I evaluated mainly the simulation part of the manuscript. Considering experiments, I do not see any significant flows or deficiencies that could diminish the value of the data and following conclusions given in the manuscript. I would even suggest improving the abstract by more explicitly saying that this work includes experimental measurements because it currently reads like purely computational work was performed.*

***We thank Reviewer #1 for the positive evaluation of our work. The abstract has now been updated to include that our work allows us to interpret existing data but also to design and perform new experimental measurements.***

*Major comments:*

*1) Although I like the central message of the paper and have no objections, I am curious whether the conclusion "a more "dynamic" or/and "mobile" part of the protein interacts with the membrane or any other (macro)(bio)molecule" makes sense globally and is not limited to LTPs. For example, it is a reasonable assumption that a more flexible part of the protein, i.e., capable of adopting necessary binding configurations, would be a more likely interacting spot. Locking in a less flexible and more specific configuration upon binding with a target molecule is also anticipated and quite typical, e.g., when ligands interact with target proteins, thereby blocking their function. The authors themselves recognize this paradigm as referring to the enzymes' dynamics.*
*It would be great if authors could comment more on dynamics-function relation, referring to the existing literature, where such observations were/were not observed for different protein families. Performing simulations on proteins that do not exhibit such a feature and do not belong to LTPs, but, e.g., structurally similar to some of the studied LTPs, would be an excellent addition too, highlighting this signature characteristic of LTPs.*

***We have now added a discussion comparing the mechanism we observe with those described for other proteins such as membrane transporters and receptors. Since those proteins are very different and have been already thoroughly characterized (including with molecular simulations) we don't think that additional simulations are required. Also, concerning protein binding dynamics, we refer to the excellent review of Wade and coworkers: "Acc. Chem. Res. 2016, 49, 5, 809–815"***

***"Notably, the conformational plasticity we observe for LTPs is reminiscent of other, previously described, functional protein mechanisms, including enzyme dynamics during catalysis (DOI: 10.1126/science.1066176), the alternating-access model of membrane transporters (https://doi.org/10.1038/nsmb.3179) or GPCR dynamics (https://doi.org/10.1021/acs.chemrev.6b00177). In all these cases, protein dynamics is strongly coupled to ligand binding (https://doi.org/10.1021/acs.accounts.5b00516) and protein function, be it for signaling, transport or enzymatic activity. Unlike for these fields, however, the contribution of structural and spectroscopic studies to uncover LTP dynamics remains quite limited, and our simulations provide an important contribution to fill this gap. We hope that our results will motivate researchers to increase efforts to experimentally quantify LTPs conformational plasticity, e.g. by structural determination of LTPs in different states (or bound to different lipids) or by single-molecule spectroscopy studies."***

*Minor comments:*

*1) Fig 1d. What is so special in Lysine compared to Arginine? Is there any disbalance in their presence in studied proteins? Any correlations between the binding affinity of certain amino acids and their overall presence on the protein surface?*

***Indeed, there is disbalance in the presence of lysine and arginine residues in our proteins. The relation between the number of these residues in our dataset is Lys:Arg = 1.6:1. On top of that, and as described in (Tubiana T et al PLoS Comput Biol. 2022 ;18(12):e1010346)***

***lysine is preferred over arginine in peripheral membrane proteins, likely because it induces fewer perturbations in the lipid bilayer. Our data also agree with Tubiana et al, concerning the correlation between abundance of specific residues on the protein surface and membrane binding.***

*2) Fig S1. GM2A and TTPA seem to be irreversibly adsorbed to the membrane on the microsecond timescale in most replicas. Is anything special in these proteins? Did this affect the sampling of a claimed membrane-binding interface?*

***Our interpretation of the different adsorption profile of GM2A and TTPA is that these two proteins appear to have higher membrane affinity in our computational assay in comparison with the other proteins in our dataset. However, this has no effect on the membrane-binding interface as the proteins are still able to undergo significant tumbling before binding to the lipid bilayer, as demonstrated by the angle between the two main protein axes and the bilayer normal before membrane binding (Fig. R1 and added as Fig.S8 to the Supplementary Information).***

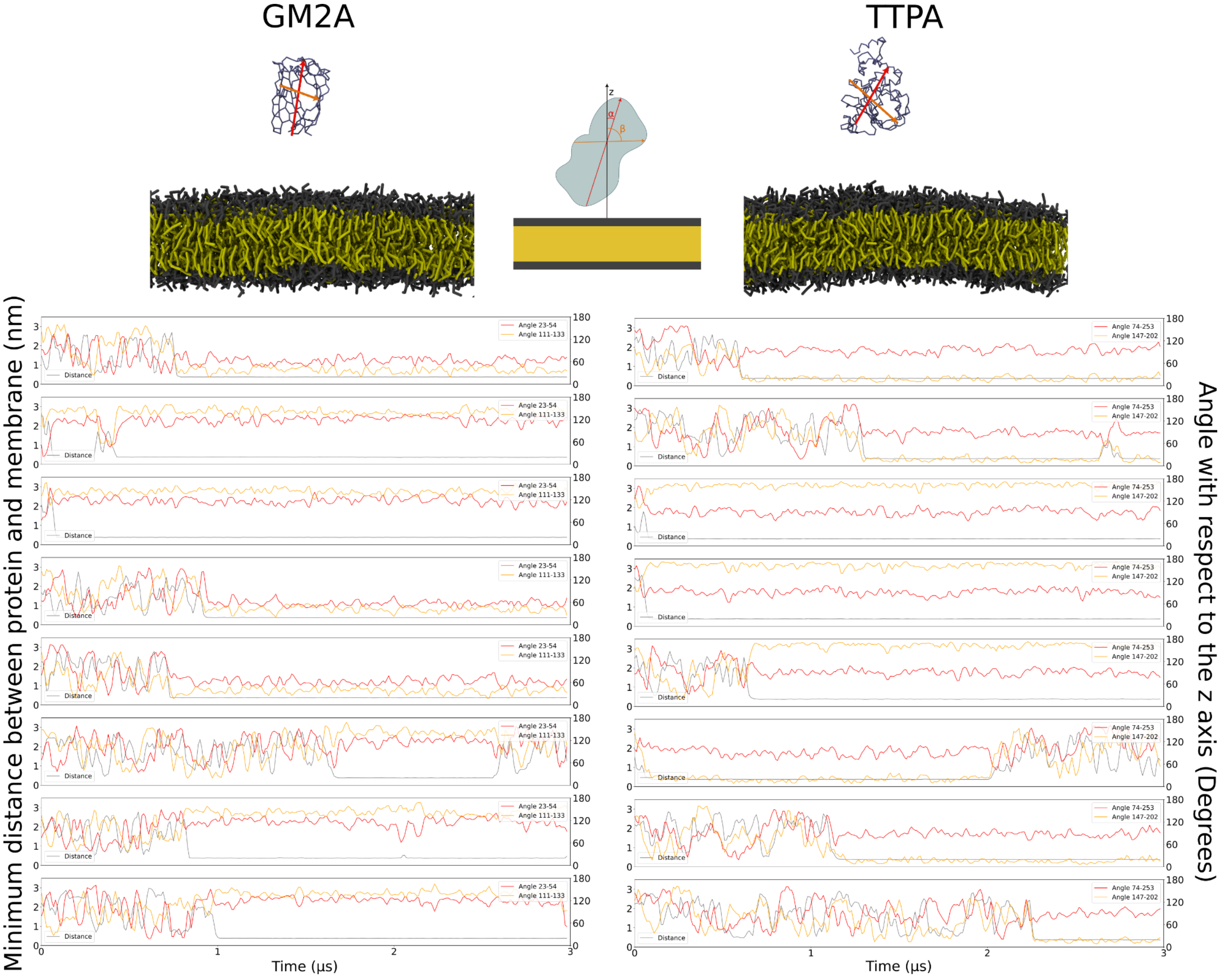

***Fig. R1. Tumbling of GM2A and TTPA in the different replicas as computed from the angle between the membrane normal and the two main protein axes (orange and red curves). As can be appreciated, the proteins undergo significant tumbling in solution before binding to membrane in most of the replicas, ultimately binding to the lipid bilayer always with the same orientation.***

*3) A related follow-up question. Multiple replicas were performed to identify the membrane-binding interface. However, if I understand well, the initial orientation of the protein with respect to the membrane was always the same. I found it a pity since performing multiple replicas starting from different initial geometries (e.g., rotating the protein in a somewhat systematic way) would likely result in a more efficient exploration of the conformation space. Can the authors comment on whether this predefined initial configuration could negatively affect the results? Performing a few additional simulations for the most problematic proteins I mentioned earlier (GM2A and TTPA) could be a nice opportunity to apply this strategy.*

***In our protocol, all proteins start from the same initial orientation but undergo significant tumbling in solution before interacting with the lipid bilayer, including for the two most extreme cases, GM2A and TTPA (Fig. R1). Hence, we think that there is no bias for what pertains to the final membrane interacting region. We have added the Fig. R1 in Supplementary Information (Fig. S8) and added the following text in the Methods Section:***

***"Despite starting from a single orientation, all proteins undergo extensive tumbling before binding to the bilayer, as illustrated by the angle between the two principal protein axes and the membrane normal for the two proteins that display the highest binding propensity, GM2A and TTPA (Fig. S8)."***

*4) How was the volume of the cavity affected by mutations in STARD11 and Mdm12? Do these data somehow correlate with the experimentally observed reduced efficiency of the lipid transfer?*

***Our data on the volume of the cavity in STARD11 and Mdm12 are inconclusive (see Figure R2 below). However, we caution from such a simplistic interpretation, since it completely neglects the lipid-bound conformation that normally has a much larger cavity than the apo form (Fig. 3).***

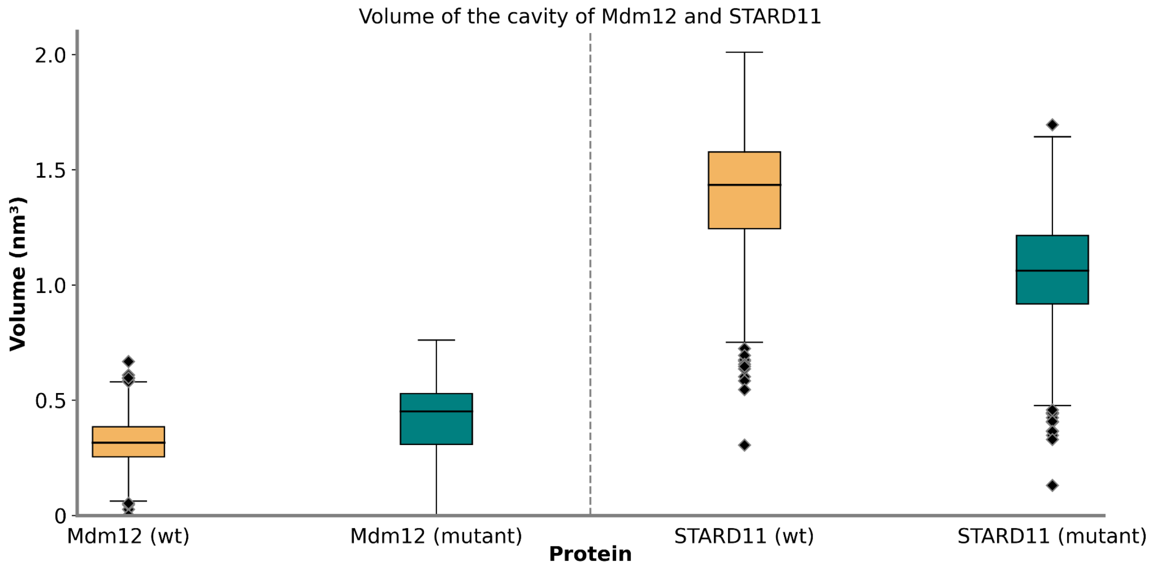


***Fig. R2. Cavity volumes for wild-type and investigated mutant forms for apo form of Mdm12 and STARD11***

*5) I would appreciate it if the authors considered playing with the templates of the main Figures at later stages because in the current version, and when printed on A4 paper, the readability of certain graphs and pictures is uncomfortable and sometimes even impossible. Obviously, the final schematics would depend on the journal and its formatting.*

***We will modify the templates of the main Figures to improve readability according to journal formatting.***

***Referees cross-commenting***

*I would like to acknowledge the thoughtful and detailed reviews provided by other reviewers. I do like their reports, and I believe that by addressing the reviewers' comments and incorporating their revisions, the article will significantly improve in terms of scientific rigor and contribution to the field.*

*Reviewer #1 (Significance (Required)):*

*This manuscript is a solid scientific work addressing gaps in our knowledge about Lipid Transfer Proteins by employing state-of-the-art methods. It advances the field on conceptual and fundamental levels. This study is of interest to both computational biophysicists and physical chemists (to whom I belong myself) as well as experimentalists, who seek a rational explanation of the experimental observations.*

***We thank the reviewer again for the positive evaluation of the significance of our work.***

# Revision Plan

*Review Commons*

***Reviewer #3*** *(Evidence, reproducibility and clarity (Required)):*
*The article "Conformational dynamics of lipid transfer domains provide a general framework to decode their functional mechanism." by Sriraksha Srinivasan, Andrea DiLuca, Arun Peter, Charlotte Gehin, Museer Lone, Thorsten Hornemann, Giovanni D'Angelo and Stefano Vanni study the interaction of Lipid transport Domains with membranes. This is done mainly by molecular modelling but also with selected experimental validations.*

*Major comments:*

*- The key conclusions are generally well supported by the analysis.*
*- The authors could however analyze in more details some aspects in which specific cases appear. For example, p3 "multiple binding and unbinding events, as shown by the minimum distance curves" does not give an entire description of the variability seen in Fig S1, e.g. LCN1 versus GM2A.*

***We now discuss in more detail the variability seen in Fig. S1 and attribute it to different membrane binding affinities of the proteins in our dataset. We also discuss how this variability could reflect the diversity of organellar membranes to which these proteins bind in vivo.***

***"Notably, the proteins in our dataset display distinct binding affinities, with some proteins showing very transient binding while others remain membrane-bound for most of the simulation trajectory (Fig. S1). This behavior could be, in part, attributed to the wide diversity of organellar membranes to which the LTDs in our dataset bind to in vivo, and to the comparative simplicity of our in silico model DOPC lipid bilayers."***

*Later the "excellent agreement" for the data in Fig S2 is not quantified which does not allow the reader to know whether it better than would have been with other methods (SASA, OPM, DREAM).*

***We have explicitly quantified this agreement by providing a direct comparison between the experimental results and our in silico assay, and we further compared it against two alternative methods: OPM and DREAMM. In detail, we have identified 12 experimentally-characterized spots suggested to be involved in membrane binding in our protein dataset (see shaded blue regions in Fig. S2). Of those 12, our method identifies all of them (100%), while DREAMM identifies 7 of them (58 %) and OPM 4 out of 8 (50 %), since of the 12 proteins we tested, only 7 are available in the OPM database. Overall, even if our approach is much noisier than the others, and thus suggesting multiple binding regions that are not currently supported by experimental observations, using physics-based methodologies appears to remain a preferable strategy to characterize the binding of peripheral proteins to lipid bilayers. Given the limited size of our dataset, we prefer not***

***to make a direct comparison between our assay and OPM/DREAMM in the main text as this won't be representative of the various methodologies.***

*p5 commenting on Fig2b the case of Osh6 that appears to disagree should probably be mentioned.*

***We now discuss this case, and attribute to this disagreement to insufficient sampling for the peculiar case of Osh6:***

***"One interesting exception in our database appears to be Osh6, where the experimentally determined membrane-binding region at the N-terminus (https://doi.org/10.1038/s41467-019-11780-y) is only marginally binding to the lipid bilayer in silico and it also appears to have limited contribution to PC1. However, our simulations are unable to sample the large conformational changes that the N-terminal lid of Osh6 has been proposed to undergo from its lipid-bound to its apo state, indicating that insufficient sampling could be the reason for this apparent discrepancy."***

*-The data and the methods are generally well presented allowing to be reproduced.*

*- The experiments adequately replicated with adequate statistical analysis.*

*Minor comments:*

*- When presenting the dataset the authors could probably detail a bit more the protocol undertaken to chose the cases. In particular it is unclear whether the chosen proteins have any membrane selectivity, which in principle could be affected by the choice of lipid used here.*

***We have now added in Table 1 a column with a list of potential organelles the different LTPs have been shown to localize to (source: UniProt). As model membrane bilayer, we opted to use a pure DOPC bilayer, for both simplicity and to compare membrane binding in a uniform setting. We foresee that future studies investigating the membrane specificity of the various proteins will shed further light into the molecular mechanism of LTPs. Finally, we also indicate that our choice of proteins was mainly driven by the availability of lipid-bound structures in the protein data bank. We have added the following sentences in the main text:***

***"Specifically, we selected all LTPs for which a crystallographic structure in complex with a lipid was available at the start of our project, plus two additional proteins (GM2A and LCN1) to increase the structural diversity of our dataset (Fig. 1a)"***

***and***

*"**Notably, the proteins in our dataset display distinct binding affinities, with some proteins showing very transient binding while other remain membrane-bound for most of the simulation trajectory (Fig. S1). This behavior could be, in part, attributed to the wide diversity of organellar membranes to which the LTDs in our dataset bind to in vivo, and to the comparative simplicity of our in silico model DOPC lipid bilayers.**"*

*- The authors could probably give some indication of how much of the variance is explained by PC1 and comment briefly on the choice to ignore other PCs.*

***PC1 explains 38 % of the total variance, on average. This means that PC1 has a large contribution to the variance, especially in comparison to the other PCs. For instance, PC2 only accounts for 17 % of the total variance. This is the reason we limited our discussion to PC1. We have added a table in supplementary Information quantifying the variance explained by PC1 and PC 2 and added the following sentence in the main text:***

***"We specifically focused on PC1 as it explains most of the variance in the dynamics (38% on average for all the proteins in our dataset). "***

*- When analyzing the residues involved in the interaction with the membrane the results could probably be compared with that of the systematic analysis performed recently: Tubiana, T., Sillitoe, I., Orengo, C., & Reuter, N. (2022). Dissecting peripheral protein-membrane interfaces. PLOS Computational Biology, 18(12), e1010346.*

***We have added in the text a reference to the work by Tubiana et al and we have further stressed that our results agree with previous observations (including theirs). This includes the preference for Lys over Arg and the importance of protruding hydrophobes:***

***"Concomitant analysis of all LTDs (Fig. 1d) indicates that the membrane binding interface of LTDs is enriched in the positively charged amino acid Lysine, as this amino acid is less membrane-disruptive than Arginine[22], and aromatic/hydrophobic ones (Phe, Leu, Val, Ile). This confirms previous observations, as (i) binding of negatively charged lipids via positively charged residues and (ii) hydrophobic insertions are two of the main mechanisms involved in membrane binding by peripheral proteins[22-27]."***

*- In the discussion on allostery/conformational selection might not be centered so much on enzymes.*

***We thank the reviewer for this important observation. We have now included in the Discussion the following paragraph that provides additional references and discussion of membrane transporters and receptors.***

***"Notably, the conformational plasticity we observe for LTPs is reminiscent of other, previously described, functional protein mechanisms, including enzyme dynamics during catalysis (DOI: 10.1126/science.1066176), the alternating-access model of membrane transporters (https://doi.org/10.1038/nsmb.3179) or GPCR dynamics (https://doi.org/10.1021/acs.chemrev.6b00177). In all these cases, protein dynamics is strongly coupled to ligand binding and protein function, be it for signaling, transport or enzymatic activity. Unlike for these fields, however, the contribution of structural and spectroscopic studies to uncover LTP dynamics remains quite limited, and our simulations provide an important contribution to fill this gap. We hope that our results will motivate researchers to increase efforts to experimentally quantify LTPs conformational plasticity, e.g. by structural determination of LTPs in different states (or bound to different lipids) or by single-molecule spectroscopy studies."***

***Reviewer #3*** *(Significance (Required)):*

*The article shows convincing results on the debated issue of the mechanism of lipid transport by lipid transfer proteins.*
*First the study employs molecular modelling to allow a rather large test on 12 cases. The molecular dynamics experiments allow the authors to draw clear hypotheses on role of protein dynamics on the interaction with membranes and the effect on bound lipids on the modification of this dynamics.*
*Then the authors use this knowledge to design experiments that largely confirm those hypotheses.*
*The results should therefore be interesting for a large audience of biochemists and cell biologists interested in lipid transport in the cell.*

***We thank Reviewer #3 for its very positive evaluation and contextualization of our work.***

## 4. Description of analyses that authors prefer not to carry out

*Please include a point-by-point response explaining why some of the requested data or additional analyses might not be necessary or cannot be provided within the scope of a revision. This can be due to time or resource limitations or in case of disagreement about the necessity of such additional data given the scope of the study. Please leave empty if not applicable.*

***We include here three of the major comments from Reviewer #2 with an accompanying explanation of the reasons why we think these requests cannot be provided within the scope of a revision.***

*• Confounding factors potentially complicate the interpretation of the in cellulo experiments. Simpler in vitro experiments may be better suited to determine if altering LTP's biophysical*

*properties, namely rationally altering the population of apo- vs holo-like configurations, quantitatively affects transport rates as suggested.*

***We agree with Reviewer #2 that this information could be useful. However, this is beyond our technical abilities, and it would require lengthy and expensive experiments that are unlikely to be completed within a reasonable time framework for a revision (3 months). We have rather opted to better discuss our model in the context of published in vitro lipid transport experiments.***

The authors highlight that a bound lipid alters LTPs' conformational ensembles akin to "conformational selection" or "induced fit." How sensitive are these findings to the bound lipid species? Do LTPs with multiple known substrates exhibit an increasing diversity of holo conformations and are different conformations stabilized by different substrates? Would similar observations (Fig 3) be made with a lipid that is not known to be transferred by a given LTP? An interesting future direction would be to examine if lipid substrate specificity could be assessed by comparing conformational ensembles to that of a known substrate and/or by overlap with the apo ensemble.

***We deem that the role of lipid specificity on LTP conformational plasticity is beyond the scope of the current work. While this topic is certainly worth future investigations, we must point out that (i) not all proteins bind/transport multiple lipids (at least according to current knowledge) and (ii) only few LTPs have been structurally characterized bound to different lipids (Osh4, Osh6, …). This limitation prevents a wide generalization, and we prefer not to speculate on this topic. So far, we have tested our approach for Osh4 bound to cholesterol or PI(4)P and found that indeed the protein exhibits different holo conformations (in agreement with the experimental data) when bound to different substrates. We have added a short comment on this topic in the Discussion section.***

***"We foresee that future studies will focus on the functional consequence of such observation, and most notably to the characterization of the extent to which such conformational changes affect multiple steps of protein function, including membrane binding or lipid extraction and release, and whether these are further modulated when different lipids are being transported."***

For LTPs to transfer lipids between membranes, transitions between apo and holo forms ought to occur when LTPs are membrane bound. How does membrane binding influence the conformational ensembles observed in solution? Does it promote conformational changes between apo- and holo-like structures, as suggested to regulate lipid uptake and release by previous studies of Osh/ORP, Ups/PRELI, and START family members? (For example: Miliara 2019 PMID 30850607, Watanabe 2015 PMID 26235513, Grabon 2017 PMID 28718450, Iaea 2015 PMID 26168008, Kudo 2008 PMID 18184806, Dong 2019 PMID 30783101) While answering these questions would require further computational effort, doing so will allow more accurate assessment of the role of conformational changes in LTP function.

**We can't unfortunately currently quantify how membrane binding influences the conformational ensembles observed in solution, as the slowdown in diffusion at the water-membrane interface makes this task computationally challenging (and certainly not feasible within the time framework of a review). We have so far tested two different proteins and have not succeeded in converging their conformational distribution when membrane-bound despite long MD simulations that lasted several months (even though the non-converged data indicate sampling of both "open" and "closed" conformations). Interestingly, our observations are in qualitative agreement with a recent study on CPTP (Rogers et al, PLOS Comp Biol, 2023), where membrane-bound CPTP is able to sample different conformations ("open" and "closed") but not to transition between the two states in 300 ns-long MD simulations.**
