# Supplementary material for: The conformational plasticity of structurally unrelated lipid transport proteins correlates with their mode of action
Source: PLoS Biol. 2024 Aug 19;22(8):e3002737. doi: 10.1371/journal.pbio.3002737 (PMC11361750; doi:10.1371/journal.pbio.3002737)
Supplement: Attachment — Submitted filename: point-by-point_19012023.pdf [file pbio.3002737.s005.pdf]

Please find below a point-by-point response to the point raised by the three reviewers.

Reviewer #1 *(Evidence, reproducibility and clarity (Required)):*

Srinivasan et al. present a comprehensive study on systematizing the structure-dynamics-function relation of lipid transfer proteins (LTPs), combining extensive molecular simulations and complementary experiments. Indeed, the current state-of-the-art in the field is quite chaotic and fractional, and such systematic studies are necessary to advance our general and conceptual understanding of the mechanisms of action of LTPs. The selected techniques and research strategies are all suitable, their description is sufficient and enables reproducibility; the obtained results are carefully presented and discussed; the conclusions are adequately supported by the data.

Given my primarily computational background, I evaluated mainly the simulation part of the manuscript. Considering experiments, I do not see any significant flows or deficiencies that could diminish the value of the data and following conclusions given in the manuscript. I would even suggest improving the abstract by more explicitly saying that this work includes experimental measurements because it currently reads like purely computational work was performed.

We thank Reviewer #1 for the positive evaluation of our work. The abstract has now been updated to include that our work allows us to interpret existing data but also to design and perform new experimental measurements.

Major comments:

1) Although I like the central message of the paper and have no objections, I am curious whether the conclusion "a more "dynamic" or/and "mobile" part of the protein interacts with the membrane or any other (macro)(bio)molecule" makes sense globally and is not limited to LTPs. For example, it is a reasonable assumption that a more flexible part of the protein, i.e., capable of adopting necessary binding configurations, would be a more likely interacting spot. Locking in a less flexible and more specific configuration upon binding with a target molecule is also anticipated and quite typical, e.g., when ligands interact with target proteins, thereby blocking their function. The authors themselves recognize this paradigm as referring to the enzymes' dynamics. It would be great if authors could comment more on dynamics-function relation, referring to the existing literature, where such observations were/were not observed for different protein families. Performing simulations on proteins that do not exhibit such a feature and do not belong to LTPs, but, e.g., structurally similar to some of the studied LTPs, would be an excellent addition too, highlighting this signature characteristic of LTPs.

We have now added a discussion comparing the mechanism we observe with those described for other proteins such as membrane transporters and receptors. Since those proteins are very different and have been already thoroughly characterized (including with

molecular simulations) we don't think that additional simulations are required. Also, concerning protein binding dynamics, we refer to the excellent review of Wade and coworkers: "Acc. Chem. Res. 2016, 49, 5, 809–815"

"The behaviour we observe for LTPs is reminiscent of other, previously described, functional protein mechanisms, including enzyme dynamics during catalysis[71], the alternating-access model of membrane transporters[72] or GPCR dynamics[73]. In all these cases, protein dynamics is strongly coupled to ligand binding[74] and protein function, be it for signaling, transport or enzymatic activity. Unlike for these fields, however, the contribution of structural and spectroscopic studies to uncover LTP dynamics remains quite limited, and our simulations provide an important contribution to fill this gap. We hope that our results will motivate researchers to increase efforts to experimentally quantify LTPs conformational plasticity, e.g. by structural determination of LTPs in different states (or bound to different lipids) or by single-molecule spectroscopy studies."

We also performed extensive analysis of protein's RMSF in relation to membrane binding and found that, like what reported for other peripheral proteins (Tubiana et al, PLoS Comput Biol. 2022 ;18(12):e1010346), disordered loops have a higher propensity for membrane binding than folded domains. We have added this text in the Results Section:

"To this end, we first characterized LTDs' local fluctuations by computing their Root Mean Square Fluctuations (RMSF), and we combined this analysis with our membrane-binding assay (Fig. 2a). Notably, we observed that almost all LTD membrane binding regions (red bars, Fig. 2b) correspond to maxima of RMSF (blue curves, Fig. 2b). To further quantify the relationship between residues' RMSF and membrane interaction frequency, we computed the RMSF for two distinct residues' populations: those that do interact with the membrane versus those that do not (Fig. 2c). Comparison of the two groups suggest that this difference is statistically significant for all proteins, with the sole exception of Sfh1. Overall, these data indicate that highly dynamical protein regions are often located at or near the membrane-binding interface regardless of the overall protein fold and structure. This observation agrees with general analyses performed on other peripheral proteins and their membrane binding motifs, which identified protruding loops as a main driver for protein-membrane interactions[35].

Minor comments:

1) Fig 1d. What is so special in Lysine compared to Arginine? Is there any disbalance in their presence in studied proteins? Any correlations between the binding affinity of certain amino acids and their overall presence on the protein surface?

Indeed, there is disbalance in the presence of lysine and arginine residues in our proteins. The relation between the number of these residues in our dataset is Lys:Arg = 1.6:1. On top of that, and as described in (Tubiana et al PLoS Comput Biol. 2022 ;18(12):e1010346) lysine is preferred over arginine in peripheral membrane proteins, likely because it induces

fewer perturbations in the lipid bilayer. Our data also agree with Tubiana et al, concerning the correlation between abundance of specific residues on the protein surface and membrane binding.

2) Fig S1. GM2A and TTPA seem to be irreversibly adsorbed to the membrane on the microsecond timescale in most replicas. Is anything special in these proteins? Did this affect the sampling of a claimed membrane-binding interface?

Our interpretation of the different adsorption profile of GM2A and TTPA is that these two proteins appear to have higher membrane affinity in our computational assay in comparison with the other proteins in our dataset. However, this has no effect on the membrane-binding interface as the proteins are still able to undergo significant tumbling before binding to the lipid bilayer, as demonstrated by the angle between the two main protein axes and the bilayer normal before membrane binding (Fig. R1 and added as Fig.S8 to the Supplementary Information).

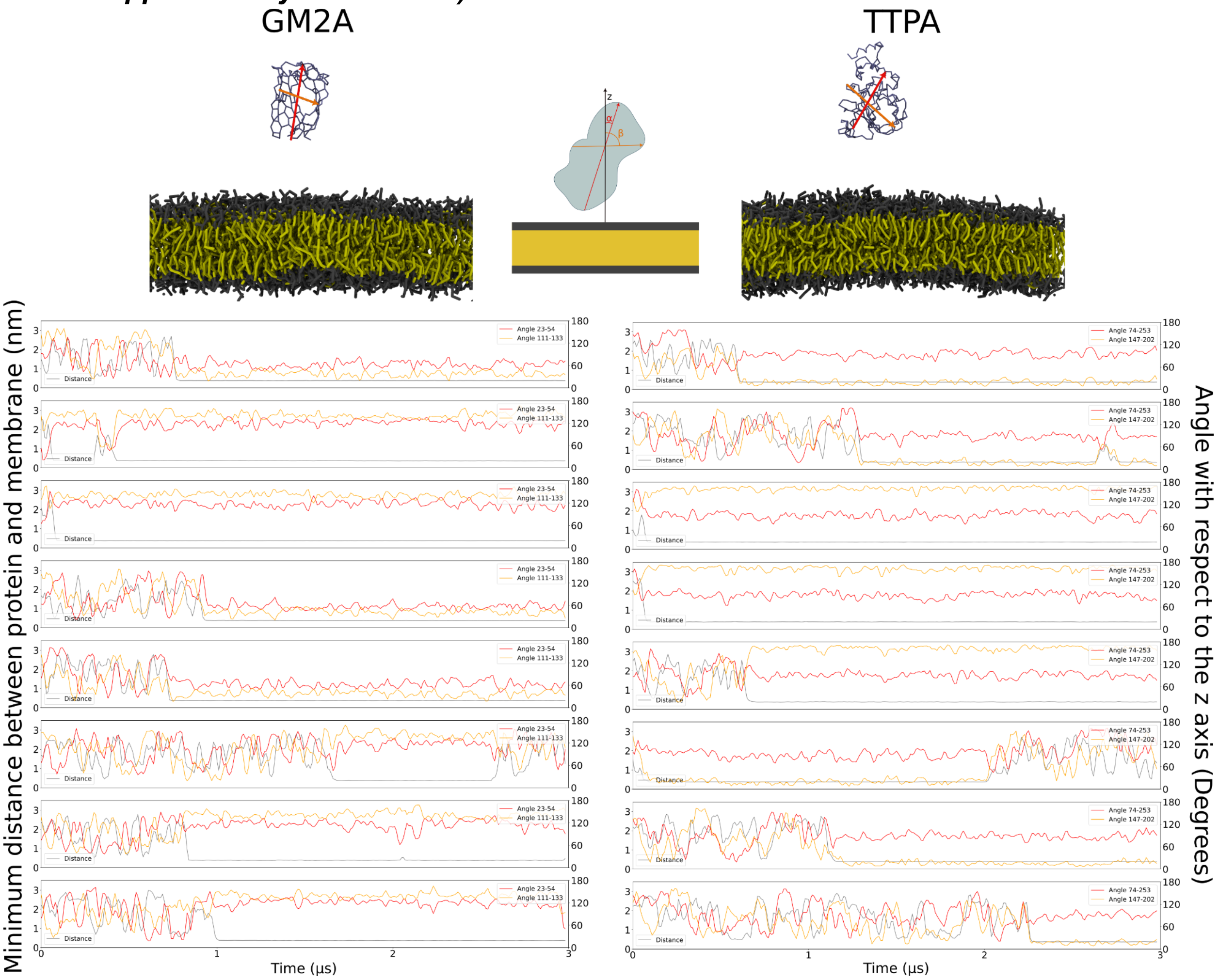

Fig. R1. Tumbling of GM2A and TTPA in the different replicas as computed from the angle between the membrane normal and the two main protein axes (orange and red curves). As

can be appreciated, the proteins undergo significant tumbling in solution before binding to membrane in most of the replicas, ultimately binding to the lipid bilayer always with the same orientation.

3) A related follow-up question. Multiple replicas were performed to identify the membrane-binding interface. However, if I understand well, the initial orientation of the protein with respect to the membrane was always the same. I found it a pity since performing multiple replicas starting from different initial geometries (e.g., rotating the protein in a somewhat systematic way) would likely result in a more efficient exploration of the conformation space. Can the authors comment on whether this predefined initial configuration could negatively affect the results? Performing a few additional simulations for the most problematic proteins I mentioned earlier (GM2A and TTPA) could be a nice opportunity to apply this strategy.

In our protocol, all proteins start from the same initial orientation but undergo significant tumbling in solution before interacting with the lipid bilayer, including for the two most extreme cases, GM2A and TTPA (Fig. R1). Hence, we think that there is no bias for what pertains to the final membrane interacting region. We have added the Fig. R1 in Supplementary Information (Fig. S8) and added the following text in the Methods Section:

"Despite starting from a single orientation, all proteins undergo extensive tumbling before binding to the bilayer, as illustrated by the angle between the two principal protein axes and the membrane normal for the two proteins that display the highest binding propensity, GM2A and TTPA (Fig. S8)."

4) How was the volume of the cavity affected by mutations in STARD11 and Mdm12? Do these data somehow correlate with the experimentally observed reduced efficiency of the lipid transfer?

Our data on the volume of the cavity in STARD11 and Mdm12 are inconclusive (see Figure R2 below). However, we caution from such a simplistic interpretation, since it completely neglects the lipid-bound conformation that normally has a much larger cavity than the apo form (Fig. 4).

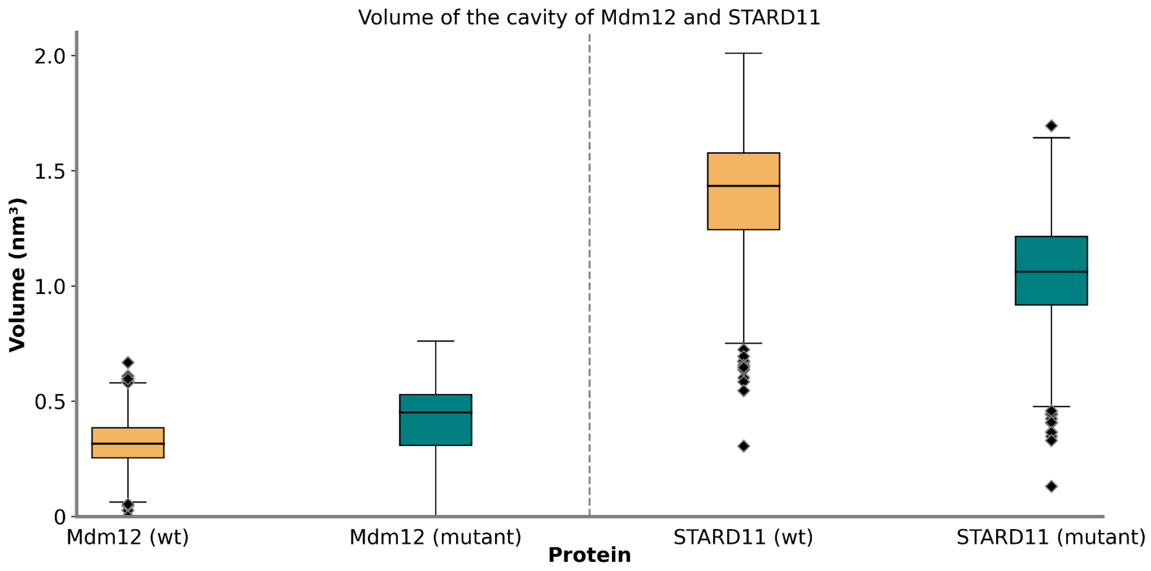

Fig. R2. Cavity volumes for wild-type and investigated mutant forms for apo form of Mdm12 and STARD11

5) I would appreciate it if the authors considered playing with the templates of the main Figures at later stages because in the current version, and when printed on A4 paper, the readability of certain graphs and pictures is uncomfortable and sometimes even impossible. Obviously, the final schematics would depend on the journal and its formatting.

We have modified the templates of the main Figures to improve readability according to journal formatting. Specifically, we have modified Figure 3 (now Figure 4) to make it easier to read as suggested by Reviewer #2.

Referees cross-commenting

I would like to acknowledge the thoughtful and detailed reviews provided by other reviewers. I do like their reports, and I believe that by addressing the reviewers' comments and incorporating their revisions, the article will significantly improve in terms of scientific rigor and contribution to the field.

Reviewer #1 (Significance (Required)):

This manuscript is a solid scientific work addressing gaps in our knowledge about Lipid Transfer Proteins by employing state-of-the-art methods. It advances the field on conceptual and fundamental levels. This study is of interest to both computational biophysicists and physical chemists (to whom I belong myself) as well as experimentalists, who seek a rational explanation of the experimental observations.

We thank the reviewer again for the positive evaluation of the significance of our work.

Reviewer #2 *(Evidence, reproducibility and clarity (Required)):*

Summary:

In a combined computational and experimental study, the authors provide insights into general features of lipid transfer proteins (LTPs), which play key roles in lipid trafficking: Through molecular dynamics simulations of a diverse set of 12 shuttle-like LTPs, they demonstrate that LTPs consistently exist in an equilibrium between two or more conformations, whose populations are modulated by a bound lipid, and that residues significantly involved in these collective conformational changes typically interact with a membrane. Their simulations indicate that conformational plasticity is a general feature of LTPs, leading them to suggest that the ability to change conformations is essential for LTP function. They test the generality of this hypothesis through in cellulo assays of two LTPs (STARD11 and Mdm12) that were not originally simulated. While experiments of STARD11 support their hypothesis, those presented for Mdm12 provide ambiguous results.

We thank Reviewer #2 for the positive evaluation of our work. In light of recent data (Wozny et al, Nature 2023) it is likely that Mdm12 does not behave mechanistically as a bona fide shuttle LTP, bur that it rather assembles as part of the ERMES complex in a soluble, non membrane-bound state. Hence, we have opted to remove the experiments on Mdm12 as their ambiguity is likely to originate from this specific mechanism that does not conform to the other LTPs in our study.

Major comments:

Throughout the manuscript, it's stated that common 'dynamical features' correlate with LTP function. The accuracy of this statement is unclear since 'dynamical features' are never precisely defined and, while equilibrium conformational ensembles are characterized, dynamics (ie kinetics or time-dependent observables) are not. Please clarify.

We have improved the scholarly presentation of our article to clarify this issue. In short, two distinct properties modulate protein function: 1. Conformational plasticity, i.e. the (thermodynamic) ability of the protein to adopt different conformations (and with different populations depending on the bound substrate). 2. Conformational "dynamics", i.e. the propensity to exchange between these different thermodynamic states. This ability depends on the free energy barriers between different states. While it is correct that we did not explicitly characterize kinetic properties (other than RMSF in this new version), principal component analysis is generally used to determine protein "essential" dynamics, i.e. to extract large conformational changes from short trajectories (see PMID 24061923 for example).
While our results hint at both aspects being relevant for lipid transport, we acknowledge that the "essential" dynamics observed from PCA analysis originates from equilibrium MD simulations, and, hence, we have opted to specifically focus on the conformational

plasticity aspect rather than the "dynamics" aspect in this revised version. This is now reflected in the new title:

"Conformational plasticity of lipid transfer domains characterizes their functional mechanism"

in the final sentences of the abstract:

"We find that the conformational landscape of LTDs display common features despite no sequence homology nor structural conservation. These features correlate with their mechanistic mode of action, allowing to interpret and design experimental strategies to further dissect their mechanism. Our findings indicate the existence of a conserved, fold-independent mechanism of lipid transfer across LTPs of various families and offer a general framework for understanding their functional mechanism."

and we have added/modified the following parts of the text:

In the Introduction:

"While these studies were limited to individual LTPs, when viewed altogether they suggest features that could be shared among many of them. Specifically, a potential role for LTP conformational plasticity, i.e. their ability to adopt multiple conformations, has been proposed for various LTPs belonging to distinct protein families, including Osh/ORP, Ups/PRELI, PITP and START family members[11, 13, 16-29]. These works have proposed that conformational changes between apo-like and holo-like structures might regulate lipid uptake and release by individual LTPs, but whether this is a general mechanism that extends to most LTPs remains unexplored.
Here, we employ a computational-based alternative approach to characterize the conformational plasticity of multiple LTDs belonging to different families. We find that despite significant differences in 3D structure, small LTDs (<50-100kDa) can adopt distinct conformations based on the presence or absence of a bound lipid. The ability to transition between these conformations has potential implications with their functional mechanism, including membrane binding, and suggests a conserved mechanism of lipid transfer across LTPs of diverse families."

As well as in several instances in the "Results" and "Discussions" Sections.

*More importantly, further evidence is needed to determine a correlation with *function*. LTPs are suggested to have faster transfer rates (a measure of function) if the apo form adopts a substantial population of holo-like conformations, akin to enzyme preorganization. This is further tested by rationally mutating STARD11 and Mdm12. However, the support for this conclusion and if these mutations alter the LTPs conformational ensembles as desired is unclear:*

As suggested by the reviewer, we have now 1. further clarified this aspect in the text and 2. generated new data that support this hypothesis (see answers below).

• Is there a quantitative correlation between the overlap of apo and holo conformational distributions (as could be quantified by KL divergence or Wasserstein distance, for example) and difference in transfer rates as suggested by Fig S6?

We have quantified the quantitative correlation between apo and holo conformational distribution for Fig.S6 (now Figure 5) and for mutant simulations using Kullback–Leibler divergence. For GRAMD1A and GRAMD1C we obtain a value of 0.71 for GRAMD1A and 3.96 for GRAMD1C, highlighting the much more similar profile of the apo-holo distributions for GRAMD1A.

• The conclusion and the generality of the findings would be greatly strengthened if a correlation can be shown for other LTPs through additional simulations of mutants whose transfer rates have been previously characterized experimentally in the literature. (For example: Ryan 2007 PMID 17344474, Grabon 2017 PMID 28718450, Iaea 2015 PMID 26168008, among many others)

In this version, we have performed analogous MD simulations of the following mutants that are defective in lipid transport rates: GRAMD1A-5P, CPTP V158N and PITP P78L. In all cases, we observe a similar behaviour to that proposed before: that overlap between apo and holo conformations is reduced in the mutants. This is now shown in new Figure 5 and the following text has been added:

"To further test this hypothesis, i.e. that overlap between apo and holo conformational populations might inform on lipid transport rates, we next identified several LTD mutants of proteins in our database that have been characterized to decrease lipid transport rates experimentally. These include: GRAMD1A 5P[47], CPTP V158N[12], and PITP P78L[48]. For these proteins, we performed MD simulations of the mutant apo form and computed the KL divergence between the apo form of these mutants and their respective wild-type holo form (Fig. 5b). Comparison between the KL divergence of the mutants with the wild type apo-holo KL divergence confirms that for all mutants, which are defective in lipid transfer, the KL divergence increases with respect to the wild-type (Fig. 5b), further suggesting that the ability to interconvert between apo and holo conformations might impact on lipid transfer rates."

• While differences in the apo conformational ensembles of the WT and mutants are observed in Fig S7b and d, if these mutations reduce overlap with holo-like conformations is not determined. Simulations of the WT holo forms are needed to properly test this hypothesis.

We have now performed these simulations and they confirm our hypothesis. The new data are shown in Fig. 6

"To alter the conformational dynamics of the START domain of STARD11, we mutated the omega loop (Fig. 6a), that is involved in the principal motion of the protein for both STARD11 (Fig. S7) as well as for the other START-containing LTDs in our dataset, GRAMD1A and GRAMD1C (Fig. 3). Analogously to what is proposed for ASTER proteins[47], we opted to mutate residues 497-501 (RVWPA, which are residues 471-475 in the crystal structure) into a stretch of prolines (for short, STARD11-5P) (Fig. 6a). As expected, this mutation results in a significant shift of the population distribution of STARD11 along the first principal component (Fig. 6b), resulting in a KL divergence shift from a value of 0.67 for the wild-type to 1.78 for the STARD11-5P mutant (Fig. 6b)."

• For Mdm12, mutations are specifically made to "lock the protein in the apo-like state;" however, the mutant adopts conformations distinct from the apo form as show in Fig S7d. How do the authors interpret the results of the cellular assays considering this and could it help explain why the mutant has similar kinetics to WT? What may explain the puzzling results of similar transfer kinetics but differing mitochondrial morphology?

As discussed above, interpretation of Mdm12 experiments could be strongly influenced by the fact that Mdm12 is part of a tunnel-like LTP complex in which it doesn't establish direct interactions with nearby organellar membranes. As such, its mechanism might be different from the one described here for other shuttle-like lipid transport domains and we have opted to remove this part entirely from the manuscript.

Confounding factors potentially complicate the interpretation of the in cellulo experiments. Simpler in vitro experiments may be better suited to determine if altering LTP's biophysical properties, namely rationally altering the population of apo- vs holo-like configurations, quantitatively affects transport rates as suggested.

As already discussed in our Revision Plan, we completely agree with Reviewer #2 that this information would be useful. However, this is beyond our technical abilities, and it would require lengthy and expensive experiments that are unlikely to be completed within a reasonable time framework for a revision (3 months). We have rather opted to better discuss our model in the context of published in vitro lipid transport experiments.

*• The abstract, intro, and title highlight that the manuscript's findings are indicative of and correlated with *function* but on p. 12 it's foreseen "that future studies will focus on the functional consequence of such observation." Please reconcile these conflicting statements and ensure connections to function are accurately described. The current title is rather bold.*

We have rewritten our title and main conclusions to clarify that while our data hint at a possible general mechanism, further functional studies will be required to confirm the correlation with *function*.

All mentions of "correlation" throughout the manuscript need to be quantitatively evaluated or

properly qualified. In addition to that mentioned above regarding Fig S6, what is the correlation coefficient between residues' contribution to PC1 and membrane interaction frequency (Fig 2)?

First, we did not observe (and discuss) correlation, but rather a direct relation: membrane binding is characterized by large motions (not the opposite way round). In addition to clarify this in the text (removing every instance of "correlation"), we have now quantified this relationship (between residues' contribution to PC1 and membrane interaction frequency) in the following way: we have split the population of residues into two distinct groups: those that do interact with the membrane versus those that do not. Then, we have compared the contributions to PC1 of these two populations. These results (now presented in Fig.3c) include a boxplot with their PC1 contributions as well as the p values resulting from a non-parametric Matt-Whitney-U-test against the null hypothesis. As can be appreciated, almost all differences are significant, with the exception of Osh6, Sfh1 and LCN1. For Osh6 and LCN1 explanations are given in the text: in short, Osh6 is missing the N-terminal lid that experiments have proposed to undergo the largest conformational change during lipid binding, while LCN1 data are extremely noisy due to its low membrane affinity in the CG simulations, resulting in a large number of membrane binding residues (and indeed reducing the threshold for membrane binding results in better correlation with PC1). Hence, Sfh1 is the only true negative results.

"We combined this analysis with our membrane-binding assay (Fig. 3), to investigate whether membrane-binding regions are also involved in the proteins' principal motions. Notably, we observed that almost all LTD membrane binding regions (red bars, Fig. 3a) correspond to maxima of the contributions of single-residue dynamics to PC1 (blue curves, Fig. 3a). As for RMSF (Fig. 2c), analysis of the relationship between residues' PC1 contribution and membrane interaction frequency for membrane interacting (Fig. 3b, orange bars) vs non-interacting (Fig. 3b, red bars) residues, indicates that membrane binding residues have significantly more contribution towards PC1 for almost all proteins (Fig. 3b). This indicates that protein regions located at or near the membrane-binding interface display large collective motions regardless of the overall protein fold and structure.
In our dataset, exceptions to this rule seem to be Sfh1, LCN1 and Osh6 (Fig. 3b). In the case of Osh6, however, the experimentally determined membrane-binding region at the N-terminus[11] is only marginally binding to the lipid bilayer in silico and it also appears to have limited contribution to PC1. This is likely because our simulations are unable to sample the large conformational changes that the N-terminal lid of Osh6 has been proposed to undergo from its lipid-bound to its apo state, indicating that insufficient sampling could be the reason for this apparent discrepancy. For LCN1, on the other hand, its low membrane affinity in CG simulations (Fig. S1) results in a large number of membrane-binding residues, effectively making this analysis very noisy."

Residue's contributions to collective conformational changes are found to be indicative of membrane binding. Yet, membrane interacting residues are identified from CG simulations that

cannot capture such collective conformational changes due to the use of an elastic network. Given that the CG simulations agree with previous experimental findings, this suggests that collective conformational changes are not important for membrane binding.

We disagree with this interpretation by Reviewer #2 of our data: we do not claim that residue's contributions to collective conformational changes are indicative of membrane binding. Rather, membrane binding happens at protein regions that display high contribution to collective conformational changes. This distinction is important: membrane binding can occur in the regions that undergo large motions, and not the other way around. Hence, protein motion does not determine membrane binding regions. In summary, it appears that, for LTPs, membrane binding regions are also characterized by collective motions (suggesting function). We have clarified this in the main text by removing all mentions of correlation, but rather discussing this specific dependency.

Are similar conclusions drawn from residues' RMSFs? In other words, are local conformational fluctuations just as indicative of membrane binding?

To test this idea, we have computed protein residues' RMSFs and analysed their relationship with membrane binding data as before: we split the population of residues into two distinct (interacting vs non-interacting) groups and we have next compared the contributions to RMSF of these two populations. Similarly to what observed before for PC1, we did observe clear relationship between local conformational fluctuations and membrane binding. We have now added these data in the text (new Figure 2), and we argued there that this observation (RMSF with membrane binding) is less surprising than that of PC1 with membrane binding. This is not unexpected and was proposed before (e.g. Tubiana et al, PLoS Comput Biol. 2022 Dec; 18(12): e1010346.). More importantly, this observation does not provide any information on lipid transport, but only on the mechanism of membrane binding. Rather, the observation of a relationship between membrane binding and global motion is more interesting, since the latter is often indicative of protein function. We have added this data in the text with the following explanation:

"To this end, we first characterized LTDs' local fluctuations by computing their Root Mean Square Fluctuations (RMSF), and we combined this analysis with our membrane-binding assay (Fig. 2a). Notably, we observed that almost all LTD membrane binding regions (red bars, Fig. 2b) correspond to maxima of RMSF (blue curves, Fig. 2b). To further quantify the relationship between residues' RMSF and membrane interaction frequency, we computed the RMSF for two distinct residues' populations: those that do interact with the membrane versus those that do not (Fig. 2c). Comparison of the two groups suggest that this difference is statistically significant for all proteins, with the sole exception of Sfh1. Overall, these data indicate that highly dynamical protein regions are often located at or near the membrane-binding interface regardless of the overall protein fold and structure. This observation agrees with general analyses performed on other peripheral proteins and their membrane binding motifs, which identified protruding loops as a main driver for protein-membrane interactions[35]"

The stated correlation may in fact be spurious and instead arise because residues at the entrance to LTP's hydrophobic cavities need to be positioned at the membrane surface for productive lipid uptake and these same residues must undergo significant conformational changes to allow lipid entry.

First, we again clarify that we did not observe (and discuss) correlation, but rather a direct relation: membrane binding is characterized by large motions (not the opposite way round). Second, this is exactly what we think it is happening and what our data show! Our combination of atomistic and CG MD simulations allows to combine two distinct observations that are hard to obtain experimentally: 1. The membrane binding interface and 2. The conformational landscape of LTDs. Our data indeed suggests that the two aspects are coupled to promote lipid uptake/release. We have clarified this aspect in the Discussion:

"Furthermore, we established that the conformational landscape of LTDs can be modulated by the bound lipid. Our results not only generalize previous observations made by us[64] and others[11, 13, 14, 16-29, 46, 65] in the context of individual LTPs, but also provide additional clues. For example, the observed relationship between membrane-binding and LTD's lipid-modulated conformational plasticity suggests that residues located at the membrane-binding interface could promote lipid entry via not only the correct positioning of the LTD hydrophobic cavity close to the membrane surface, but also by undergoing specific conformational changes to promote lipid uptake/release. We foresee that future studies will further investigate the functional consequence of such observation, and most notably the characterization of the extent to which such conformational changes affect multiple steps of lipid transport, including membrane binding or lipid extraction and release, and whether these are further modulated when different lipids are being transported."

Is proximity to cavity entrance more or less correlated with membrane binding than 'dynamics'?

It is likely that proximity to cavity entrance would correlate with membrane binding more than "dynamics". However, we have to consider that we do not know where the cavity entrance in LTPs is located simply based on structure alone, (we can often only infer it from the structure). To this end, we have recently published a distinct manuscript in BioRxiv where we propose a new methodology to investigate lipid entry into LTPs (Srinivasan et al, BioRxiv, 2023.11.24.568550), and, although not explicitly stated, we generally observe good correlation with membrane binding as computed using CG simulations.

p. 12 speculatively suggests "the high degree of protein dynamics we observed in membrane proximal regions could potentially facilitate the energetically unfavorable reaction that involves the extraction of a lipid from a membrane." Yet, the logic behind this idea does not make sense since

a free energy barrier, an equilibrium thermodynamic quantity, cannot be lowered by changes in dynamics. Please explain.

We have now clarified that the high degree of protein "dynamics" refers to RMSF and PC analysis, and we have also clarified our underlying (speculative) hypothesis: while our current understanding of the mechanism of lipid extraction is quite poor, both using chemical intuition and following a recent MD study on one LTP (Rogers et al, 2023, Plos Comp Biol), it is safe to assume that the hydrophobic environment around the lipid is important for its stabilization in the lipid bilayer. Hence, reducing the number of hydrophobic contacts between the lipid and its environment could facilitate transport. A highly dynamic protein, by cycling between different conformations, could "stir" the bilayer, and hence decrease the number of contacts between the lipid and its environment favouring transport. We have added the following text in the Discussion Section:

"However, we found that across LTDs, the interface and/or residues adjacent to it exhibited high fluctuations in solution, and that they displayed the largest collective motion according to principal component analysis. This observation hints that irrespective of the nature of the transported lipid, the high degree of protein conformational plasticity we observed in membrane proximal regions could potentially facilitate the energetically unfavorable extraction of a lipid from a membrane and its transfer to the hydrophobic groove of a LTP. This process has also been proposed as the rate-limiting step for lipid transport[62], and a recent study on CPTP has proposed that lipid transport is enhanced by the disruption of hydrophobic lipid-membrane contacts[63]. Based on our data, it is tempting to speculate that the ability of LTDs to adopt multiple conformations could further contribute to a reduction in lipid-membrane contacts and, hence, lipid transport. However, the slow-down in diffusion at the water-membrane interface makes it computationally challenging to investigate protein conformational transitions when adsorbed on the membrane.."

Examining how the LTPs impact membrane properties would offer insight into the functional relevance of such residues for lipid extraction.

To address this point, we have performed atomistic simulations of GM2A in its membrane-bound state to compute hydrophobic contacts in the bilayer as proposed in (Rogers et al, 2023, Plos Comp Biol). We observed that there is a clear depletion of hydrophobic contacts in the protein surroundings. We include below results from 3 different atomistic simulations, backmapped from different replicas of the CG simulations. The results from a protein-free bilayer as a negative control are also included in the figure:

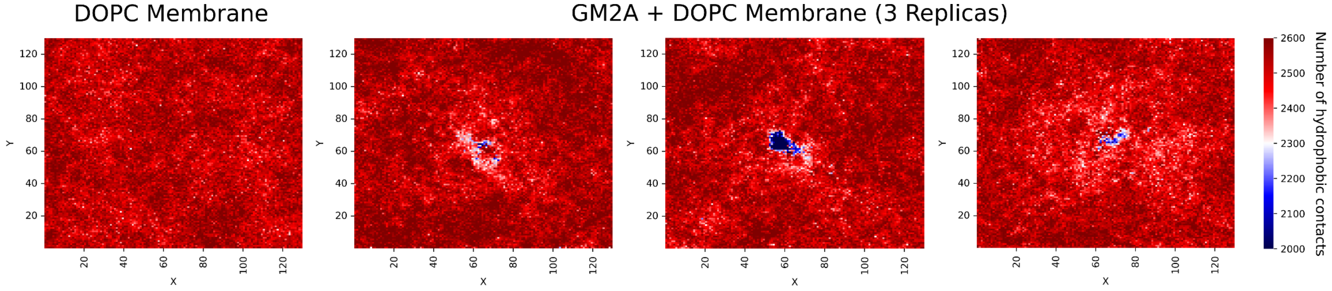

For comparison, this is a result from CG simulations, in qualitative agreement with the AA simulations, although the differences are more subtle:

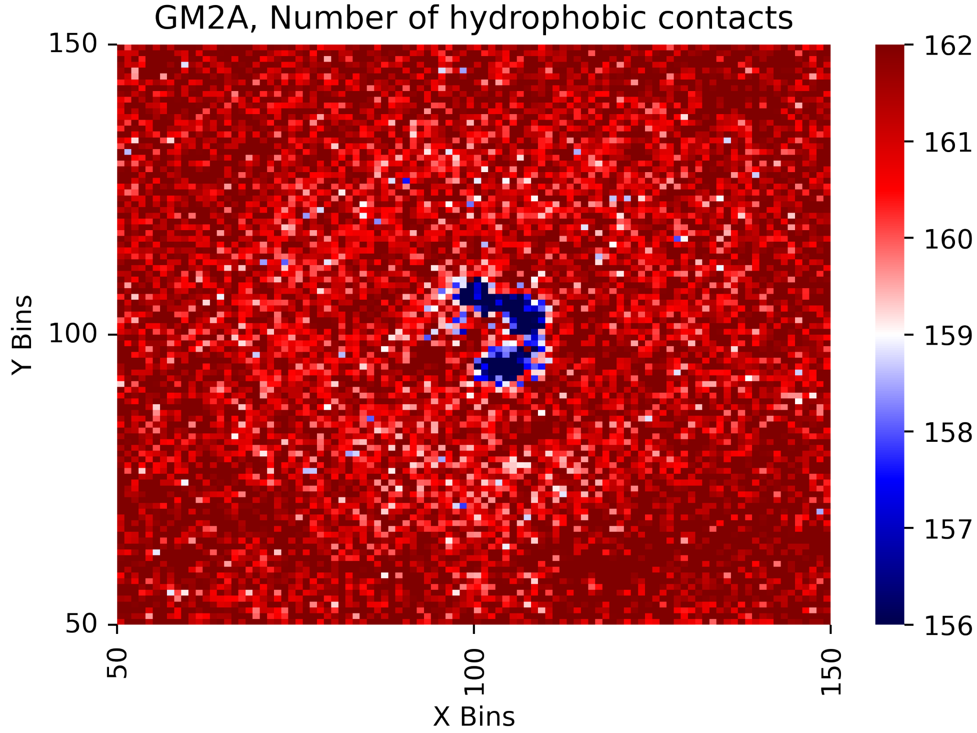

However, since we could carry out these simulations for only one protein during the revision timeframe, we opted to simply refer to the recent existing article by Rogers et al in the Discussion section:

"However, we found that across LTDs, the interface and/or residues adjacent to it exhibited high fluctuations in solution, and that they displayed the largest collective motion according to principal component analysis. This observation hints that irrespective of the nature of the transported lipid, the high degree of protein conformational plasticity we observed in membrane proximal regions could potentially facilitate the energetically unfavorable extraction of a lipid from a membrane and its transfer to the hydrophobic groove of a LTP. This process has also been proposed as the rate-limiting step for lipid

transport[62], and a recent study on CPTP has proposed that lipid transport is enhanced by the disruption of hydrophobic lipid-membrane contacts[63]. Based on our data, it is tempting to speculate that the ability of LTDs to adopt multiple conformations could further contribute to a reduction in lipid-membrane contacts and, hence, lipid transport. However, the slow-down in diffusion at the water-membrane interface makes it computationally challenging to investigate protein conformational transitions when adsorbed on the membrane."

The authors highlight that a bound lipid alters LTPs' conformational ensembles akin to "conformational selection" or "induced fit." How sensitive are these findings to the bound lipid species? Do LTPs with multiple known substrates exhibit an increasing diversity of holo conformations and are different conformations stabilized by different substrates? Would similar observations (Fig 3) be made with a lipid that is not known to be transferred by a given LTP? An interesting future direction would be to examine if lipid substrate specificity could be assessed by comparing conformational ensembles to that of a known substrate and/or by overlap with the apo ensemble.

We deem that the role of lipid specificity on LTP conformational plasticity is beyond the scope of the current work. While this topic is certainly worth future investigations, we must point out that (i) not all proteins bind/transport multiple lipids (at least according to current knowledge) and (ii) only few LTPs have been structurally characterized bound to different lipids (Osh4, Osh6, …). This limitation prevents a wide generalization, and we prefer not to speculate on this topic. So far, we have tested our approach for Osh4 bound to cholesterol or PI(4)P and found that indeed the protein exhibits different holo conformations (in agreement with the experimental data) when bound to different substrates. We have added a short comment on this topic in the Discussion section.

" We foresee that future studies will further investigate the functional consequence of such observation, and most notably the characterization of the extent to which such conformational changes affect multiple steps of lipid transport, including membrane binding or lipid extraction and release, and whether these are further modulated when different lipids are being transported."

For LTPs to transfer lipids between membranes, transitions between apo and holo forms ought to occur when LTPs are membrane-bound. How does membrane binding influence the conformational ensembles observed in solution? Does it promote conformational changes between apo- and holo-like structures, as suggested to regulate lipid uptake and release by previous studies of Osh/ORP, Ups/PRELI, and START family members? (For example: Miliara 2019 PMID 30850607, Watanabe 2015 PMID 26235513, Grabon 2017 PMID 28718450, Iaea 2015 PMID 26168008, Kudo 2008 PMID 18184806, Dong 2019 PMID 30783101) While answering these questions would require further computational effort, doing so will allow more accurate assessment of the role of conformational changes in LTP function.

We can't unfortunately currently quantify how membrane binding influences the conformational ensembles observed in solution, as the slowdown in diffusion at the water-membrane interface makes this task computationally challenging (and certainly not feasible within the time framework of a review). We have so far tested two different proteins and have not succeeded in converging their conformational distribution when membrane-bound despite long MD simulations that lasted several months (even though the non-converged data indicate sampling of both "open" and "closed" conformations). Interestingly, our observations are in qualitative agreement with a recent study on CPTP (Rogers et al, PLOS Comp Biol, 2023), where membrane-bound CPTP is able to sample different conformations ("open" and "closed") but not to transition between the two states in 300 ns-long MD simulations. We have added the following text in the Discussion:

"However, we found that across LTDs, the interface and/or residues adjacent to it exhibited high fluctuations in solution, and that they displayed the largest collective motion according to principal component analysis. This observation hints that irrespective of the nature of the transported lipid, the high degree of protein conformational plasticity we observed in membrane proximal regions could potentially facilitate the energetically unfavorable extraction of a lipid from a membrane and its transfer to the hydrophobic groove of a LTP. This process has also been proposed as the rate-limiting step for lipid transport[62], and a recent study on CPTP has proposed that lipid transport is enhanced by the disruption of hydrophobic lipid-membrane contacts[63]. Based on our data, it is tempting to speculate that the ability of LTDs to adopt multiple conformations could further contribute to a reduction in lipid-membrane contacts and, hence, lipid transport. However, the slow-down in diffusion at the water-membrane interface makes it computationally challenging to investigate protein conformational transitions when adsorbed on the membrane."

*The authors motivate the study with the *assumption* that a common molecular mechanism of LTP function exists. Yet LTPs have evolved diverse sequences, structures, and substrate preferences; thus there seems to be no a priori requirement (or even necessarily a benefit) for a single molecular mechanism. What evidence then supports this premise? While previous studies are limited to individual LTPs, when viewed altogether retrospectively, they suggest features that could be shared among LTPs. Synthesizing previous studies and more thoroughly referencing them (only 5 are cited in the intro on p. 3) would strengthen both the premise and findings of the manuscript.*

Indeed, despite having different structures, substrates and the ability to target distinct organelles, previous evidence on LTPs seem to suggest a potential role for protein conformational plasticity for function, e.g. for Osh/ORP (Jun Im et al, Nature 2005; Canagarajah et al, JMB 2008; Moser von Filseck et al, Nat Comm, 2015; Lipp et al, Nat Comm. 2019,...), StART (Arakane et al, PNAS, 1996; Feng et al, Biochemistry, 2000; Grabon et al, JBC, 2017; Khelashvili et al, eLife, 2019;...) Ups/PRELI (Miliara 2019 PMID 30850607, Watanabe 2015 PMID 26235513,) and PITP domains (Tremblay et al, Archives of

Biochemistry and Biophysics, 2005; Ryan et al, MBOC, 2007; Grabon 2017 PMID 28718450). Our simulations provide additional evidence in this direction, allowing to draw parallelisms with "enzyme-like" or transporter-like" features that could be exploited for further design of testable hypotheses. We have rewritten our introduction to better contextualize/acknowledge previous findings and to clarify these points:

"While these studies were limited to individual LTPs, when viewed altogether they suggest features that could be shared among many of them. Specifically, a potential role for LTP conformational plasticity, i.e. their ability to adopt multiple conformations, has been proposed for various LTPs belonging to distinct protein families, including Osh/ORP, Ups/PRELI, PITP and START family members[11, 13, 16-29]. These works have proposed that conformational changes between apo-like and holo-like structures might regulate lipid uptake and release by individual LTPs, but whether this is a general mechanism that extends to most LTPs remains unexplored."

The LTPs investigated are known to target distinct membranes. Should they then be expected to share structural or sequence-based features predictive of membrane binding interfaces, as motivates the analysis in Fig 1d, 1e, and S3? Or is it beneficial for LTPs to recognize membranes in different ways?

Since membrane binding is membrane/organelle-specific, it is possible that residue's diversity in membrane binding interfaces could indeed be beneficial for this diversity. We have added this cautionary comment as a potential explanation of our finding of a lack of conserved sequence-based features for membrane binding interfaces:

"However, analysis on a protein-by-protein scale reveals a lack of any general trend, as each protein family displays a characteristic amino acid composition in its membrane binding interface (Fig. S3), possibly as a consequence of the diversity of the organellar membrane they bind to (Table 1)."

Minor comments:

p. 2 "making lipid transfer across the cytoplasm a potentially energetically favorable process": Is it meant that it is less energetically costly than transfer without a LTP? Why it would be energetically favorable is unclear (and would indicate that the LTP sequesters lipids away from membranes instead of transferring them between membranes).

Yes, this is what we meant. We have rewritten this appropriately:

"Despite growing interest in the non-vesicular lipid transport pathway, our mechanistic understanding of how LTPs perform their function is still largely incomplete. The only unifying feature of LTPs is a polar exterior and the presence of a lipid transfer domain (LTD) containing a hydrophobic cavity that encloses the lipid. This architecture, by

shielding the hydrophobic lipid molecule from the aqueous environment of the cytoplasm, reduces the energetic cost of transferring a lipid across two membranes.”

p. 3 "The excellent agreement between the membrane interface determined from the simulations and the experimentally-proposed one available for... Osh6" is missing a citation.

We have now added the relevant citation.

The plots in Fig 1d and S3 are difficult to interpret. Bar plots, for example, would allow easier comparison and evaluation. Currently, it seems that most proteins individually exhibit some of the same trends observed among the whole set, counter to the conclusion on p 5.

We have modified and improved the presentation of Figures 1d according to the reviewer’s comment.

Negatively charged residues engage in a number of membrane interactions (Fig 1d and S3). What is a potential explanation for this unconventional observation?

One possible interpretation is that negatively charged residues could interact with positively charged moieties (ethanolamine, choline) of PC and PE lipids.

How much variance is captured by PC1, and how many PCs are needed to capture most of the variance in the conformations?

PC1 explains 38 % of the total variance, by average, whereas PC2 accounts for 17 % of it. Therefore, PC1 and PC2 capture most of the variance in almost all cases.
We have also added this to the text:

"We specifically focused on PC1 as it explains most of the variance in the dynamics (38% on average for all the proteins in our dataset, see Supplementary Table 1)."

We have computed this variance and we have added this analysis in Supplementary Information.

Plots in Fig 3, especially panels c and d are difficult to see. Please make the panels larger (perhaps a 3 x 4 layout instead of 2 x 6 would work better).

We have improved the presentation of Figure 3 following this suggestion.

p. 8 "these conformational changes are localized in protein regions that interact with the lipid bilayer" is contradicted by the results in Fig 2b showing that all residues with large contributions to PC1 do not interact with the membrane and discussed on p 5.

As discussed above, we don't observe "correlation" between membrane binding and conformational plasticity, but we rather observe that membrane binding regions display high conformational plasticity (the opposite is not true). We have clarified this in the text.

p. 8 "in the absence of bound lipids, it is able to sample multiple conformations" is not supported by the orange distributions in Fig 3d that appear unimodal. Is it instead meant that the apo form exhibits larger variance in cavity volume?

Yes, this is what we meant. We have clarified this in the text:

"Consistent with the behavior of the entire protein (Fig. 4c), the cavity in the holo state generally adopts a more compact distribution of conformations (Fig. S6, purple), while, in the absence of bound lipids, the cavity exhibits larger variance in its volume (Fig. S6, orange)."

Please clarify if the elastic network was constructed to maintain the holo or apo structures of each protein and if a bound lipid was used in the CG simulations.

For membrane binding CG simulations, we used the apo structure and no bound lipid was used in the simulations. However, analogous simulations in the holo form (not shown) have essentially identical membrane binding interfaces. We have added this clarification in the Methods:

"An additional elastic network with a force constant of 700 kJ mol^{-1} nm^{-2}, and upper elastic bond cut-off of 0.8 nm, was used to restrain the secondary structure of the protein in its apo structure."

*Was *CHARMM* TIP3P used?*

Yes. We have now specified this in the methods section:

"A cubic box of edge length was constructed such that the distance between the protein and the edge of the box was at least 1 nm, and the systems were solvated with CHARMM TIP3P water and ionized with 0.12M of sodium and chloride ions."

Please clarify how membrane interacting residues were defined and how interaction frequency was calculated from the longest duration of interaction.

We have added this explanation in the Methods:

"The frequency of interaction with the membrane for each residue as shown in Fig. 2b and Fig. 3a corresponds to the normalized longest duration of interaction for that residue,

obtained from the Prolint package, with a cutoff distance of 7 Å used to define the protein-lipid interactions. The normalized binding frequencies for each protein were obtained by dividing all the longest duration of interaction data by its corresponding largest value. Similarly, the frequency of interaction for each amino acid and each secondary structure type in the LTD (Fig. 1d, 1e) were determined by summing the longest duration of interaction for each amino acid/secondary structure type within the protein and obtaining normalized values between 0 and 1 for every protein. The mean interaction across all proteins was then determined by averaging over these normalized values."

Refs 16 and 45 refer to the same paper.

Thanks, it is now corrected!

***Reviewer #2** (Significance (Required)):*

General assessment:

The work aims to tackle a grand question regarding membrane homeostasis mechanisms-what are universal principles underlying LTP function-and offers initial insights; however, further evidence is needed to support the conclusions as written, and some key results require further investigation and explanation.

Advance and audience:

By concurrently investigating the largest number of lipid transfer proteins to-date, the authors provide data invaluable for uncovering general mechanisms of non-vesicular lipid transport and advancing our understanding of membrane homeostasis mechanisms. By illuminating the wide-spread importance of conformational plasticity among lipid transfer proteins, the work presents a conceptual advance in our understanding of lipid transfer mechanisms and unifies previous studies. Because the manuscript emphasizes common biophysical principles and draws connections to enzyme biophysics, it ought to be of interest not only to membrane biologists but biochemists and molecular biologists more broadly.

We thank Reviewer #2 for the very positive evaluation of the significance of our work and for the in-depth analysis provided that has certainly helped improve the quality of our work.

***Reviewer #3** (Evidence, reproducibility and clarity (Required)):*
The article "Conformational dynamics of lipid transfer domains provide a general framework to decode their functional mechanism." by Sriraksha Srinivasan, Andrea DiLuca, Arun Peter, Charlotte Gehin, Museer Lone, Thorsten Hornemann, Giovanni D'Angelo and Stefano Vanni study the interaction of Lipid transport Domains with membranes. This is done mainly by molecular modelling but also with selected experimental validations.

Major comments:

- The key conclusions are generally well supported by the analysis.
- The authors could however analyze in more details some aspects in which specific cases appear. For example, p3 "multiple binding and unbinding events, as shown by the minimum distance curves" does not give an entire description of the variability seen in Fig S1, e.g. LCN1 versus GM2A.

We now discuss in more detail the variability seen in Fig. S1 and attribute it to different membrane binding affinities of the proteins in our dataset. We also discuss how this variability could reflect the diversity of organellar membranes to which these proteins bind in vivo.

"Notably, the proteins in our dataset display distinct binding affinities, with some proteins showing very transient binding while others remain membrane-bound for most of the simulation trajectory (Fig. S1). This behavior could be, in part, attributed to the wide diversity of organellar membranes to which the LTDs in our dataset bind to in vivo, and to the comparative simplicity of our in silico model lipid bilayers."

Later the "excellent agreement" for the data in Fig S2 is not quantified which does not allow the reader to know whether it better than would have been with other methods (SASA, OPM, DREAM).

We have explicitly quantified this agreement by providing a direct comparison between the experimental results and our in silico assay, and we further compared it against two alternative methods: OPM and DREAMM. In detail, we have identified 12 experimentally-characterized spots suggested to be involved in membrane binding in our protein dataset (see shaded blue regions in Fig. S2). Of those 12, our method identifies all of them (100%), while DREAMM identifies 7 of them (58 %) and OPM 4 out of 8 (50 %), since of the 12 proteins we tested, only 8 are available in the OPM database. Overall, even if our approach is much noisier than the others, and thus suggesting multiple binding regions that are not currently supported by experimental observations, using physics-based methodologies appears to remain a preferable strategy to characterize the binding of peripheral proteins to lipid bilayers. Given the limited size of our dataset, we prefer not to make a direct comparison between our assay and OPM/DREAMM in the main text as this won't be representative of the various methodologies.

p5 commenting on Fig2b the case of Osh6 that appears to disagree should probably be mentioned.

We now discuss this case, and attribute to this disagreement to insufficient sampling for the peculiar case of Osh6:

"In the case of Osh6, however, the experimentally determined membrane-binding region at the N-terminus[11] is only marginally binding to the lipid bilayer in silico and it also appears to have limited contribution to PC1. This is likely because our simulations are unable to sample the large conformational changes that the N-terminal lid of Osh6 has been proposed to undergo from its lipid-bound to its apo state, indicating that insufficient sampling could be the reason for this apparent discrepancy."

-The data and the methods are generally well presented allowing to be reproduced.

- The experiments adequately replicated with adequate statistical analysis.

Minor comments:

- When presenting the dataset the authors could probably detail a bit more the protocol undertaken to chose the cases. In particular it is unclear whether the chosen proteins have any membrane selectivity, which in principle could be affected by the choice of lipid used here.

We have now added in Table 1 a column with a list of potential organelles the different LTPs have been shown to localize to (source: UniProt). As a model membrane bilayer, we opted to use a pure DOPC bilayer, for both simplicity and to compare membrane binding in a uniform setting. We foresee that future studies investigating the membrane specificity of the various proteins will shed further light on the molecular mechanism of LTPs. Finally, we also indicate that our choice of proteins was mainly driven by the availability of lipid-bound structures in the protein data bank. We have added the following sentences in the main text:

"Specifically, we selected all LTPs for which a crystallographic structure in complex with a lipid was available at the start of our project, plus two additional proteins (GM2A and LCN1) to increase the structural diversity of our dataset (Fig. 1a)"

and

"Notably, the proteins in our dataset display distinct binding affinities, with some proteins showing very transient binding while others remain membrane-bound for most of the simulation trajectory (Fig. S1). This behavior could be, in part, attributed to the wide diversity of organellar membranes to which the LTDs in our dataset bind to in vivo, and to the comparative simplicity of our in silico model lipid bilayers."

- The authors could probably give some indication of how much of the variance is explained by PC1 and comment briefly on the choice to ignore other PCs.

PC1 explains 38 % of the total variance, on average. This means that PC1 has a large contribution to the variance, especially in comparison to the other PCs. For instance, PC2 only accounts for 17 % of the total variance. This is the reason we limited our discussion to PC1. We have added a table in supplementary Information quantifying the variance explained by PC1 and PC 2 and added the following sentence in the main text:

"We specifically focused on PC1 as it explains most of the variance in the dynamics (38% on average for all the proteins in our dataset)."

- When analyzing the residues involved in the interaction with the membrane the results could probably be compared with that of the systematic analysis performed recently: Tubiana, T., Sillitoe, I., Orengo, C., & Reuter, N. (2022). Dissecting peripheral protein-membrane interfaces. PLOS Computational Biology, 18(12), e1010346.

We have added in the text a reference to the work by Tubiana et al and we have further stressed that our results agree with previous observations (including theirs). This includes the preference for Lys over Arg and the importance of protruding hydrophobes:

"Concomitant analysis of all LTDs (Fig. 1d) indicates that the membrane binding interface of LTDs is enriched in the positively charged amino acid Lysine, as this amino acid is less membrane-disruptive than Arginine[35], and aromatic/hydrophobic ones (Phe, Leu, Val, Ile). This confirms previous observations, as (i) binding of negatively charged lipids via positively charged residues and (ii) hydrophobic insertions are two of the main mechanisms involved in membrane binding by peripheral proteins[35-40]. "

- In the discussion on allostery/conformational selection might not be centered so much on enzymes.

We thank the reviewer for this important observation. We have now included in the Discussion the following paragraph that provides additional references and discussion of membrane transporters and receptors.

"The behaviour we observe for LTPs is reminiscent of other, previously described, functional protein mechanisms, including enzyme dynamics during catalysis[71], the alternating-access model of membrane transporters[72] or GPCR dynamics[73]. In all these cases, protein dynamics is strongly coupled to ligand binding[74] and protein function, be it for signaling, transport or enzymatic activity. Unlike these fields, however, the contribution of structural and spectroscopic studies to uncover LTP dynamics remains quite limited, and our simulations provide an important contribution to fill this gap. We hope that our results will motivate researchers to increase efforts to experimentally quantify LTPs conformational plasticity, e.g. by structural determination of LTPs in different states (or bound to different lipids) or by single-molecule spectroscopy studies."

Reviewer #3 *(Significance (Required)):*

The article shows convincing results on the debated issue of the mechanism of lipid transport by lipid transfer proteins.
First the study employs molecular modelling to allow a rather large test on 12 cases. The molecular dynamics experiments allow the authors to draw clear hypotheses on role of protein dynamics on the interaction with membranes and the effect on bound lipids on the modification of this dynamics.
Then the authors use this knowledge to design experiments that largely confirm those hypotheses.
The results should therefore be interesting for a large audience of biochemists and cell biologists interested in lipid transport in the cell.

We thank Reviewer #3 for the very positive evaluation and contextualization of our work.
