# Supplementary material for: The conformational plasticity of structurally unrelated lipid transport proteins correlates with their mode of action
Source: PLoS Biol. 2024 Aug 19;22(8):e3002737. doi: 10.1371/journal.pbio.3002737 (PMC11361750; doi:10.1371/journal.pbio.3002737)
Supplement: Attachment — Submitted filename: Response_Reviewers_4.docx [file pbio.3002737.s006.docx]

Dear Editor, Reviewers,

please find below our answers to the reviewers’ comments. We have now addressed all main points and specifically we have:

1. added simulations of the mutant-holo version and critically discussed these results
2. addressed all minor concerns and improved the discussion on all points raised by the reviewers

We want to thank the reviewers for their contribution towards improving the quality of our manuscript. We also want to stress that we firmly believe that PLOS biology (rather than PLOS Computational Biology) is the appropriate platform for our work, not only because we use both computational and experimental approaches, but also because our results will be of interest to both computational and experimental scientists working on lipid transport.

Sincerely and on behalf of all authors,

Stefano Vanni

Reviewers' comments

Rev. 1:

Having previously reviewed this manuscript positively (Reviewer 1 in the first iteration), my current feedback will be concise and focused.

Srinivasan et al. present a comprehensive study on the structure-dynamics-function relationship of lipid transfer proteins through extensive molecular simulations complemented by some experimental validation.

The research is both timely and significant.

The methodologies and strategies employed are well-chosen and described in sufficient detail for reproducibility.

The results are clearly presented and thoughtfully discussed.

The conclusions drawn are supported by the evidence.

The revisions, mainly prompted by feedback from the review process, have notably enhanced the manuscript, with deeper simulation analyses adding substantial value.

While, in principle, I endorse the acceptance of this article in its present form, I offer two points for consideration:

1. Journal Suitability

Although the manuscript has been strengthened through revisions, it has shifted its focus towards computational analysis, particularly following the removal of experimental data on Mdm12 due to the non-mechanistic signature in its functionality.

This shift renders the study more aligned with computational biology, suggesting PLOS Computational Biology as a potentially more fitting publication venue.

Though I proceed with a "Minor Revision" recommendation (the system does not allow me to offer another suitable recommendation unambiguously), I highlight this consideration regarding journal alignment for the authors' and editors' contemplation.

While we understand this reviewer position, we want to stress that we opted to submit this manuscript to PLOS biology not as a consequence of the techniqes we used, but rather based on the audience we want to target. We believe that our findings will be of interest to a large audience of both experimentalists and computational scientists interested in lipid transport proteins. As such, we firmly believe that PLOS biology is the appopriate journal for our study.

2. Clarification on Simulation-Experiment Agreement

There appears to be a contradiction in the manuscript regarding the agreement between simulations and experimental findings, particularly concerning the membrane-binding interfaces.

While initial statements highlight an "excellent agreement" (page 5, last paragraph), subsequent discussion regarding Osh6 (page 10) indicates discrepancies potentially attributed to simulation limitations in capturing extensive conformational changes.

I suggest the authors refine their narrative to accurately reflect these nuances and ensure consistency in their discussion of simulation-experiment agreement.

We have further clarified simulation limitations in the context of membrane binding and offered a nuanced description of our computational results regarding membrane binding:

“**Despite intrinsic limitations of our CG simulations for what pertains the description of protein conformational changes**, the **good** agreement between the membrane interface determined from the simulations and the experimentally-proposed one available for CPTP^31^, FABP^32^, Osh4^21^, Osh6^11^ GRAMD1A^27^, GRAMD1C^27^, PITPA^33^, and TTPA^34^ (Fig. S2) suggests that our *in silico* methodology is **mostly** able to reproduce the correct membrane-binding interface of LTDs, similar to what was shown for different families of membrane-binding peripheral proteins^30^.”

Rev. 2:

The authors have substantially improved the manuscript through their revisions. I have one remaining concern that needs to be addressed before I can support publication and a few minor comments/suggestions:

My main concern regards the investigations of newly added mutants (GRAM1DA-5P, CPTP V158N, PITPA P78L) and STARD11-5P mutant to test the hypothesis that overlap between apo and holo conformational distributions informs on lipid transport rates. To test this hypothesis, the conformational distributions of mutant-apo and mutant-holo should be compared and their overlap found to be smaller than the corresponding overlap between wt-apo and wt-holo distributions. Currently the mutant-apo is compared to the wt-holo distribution; however, this does not rule out the possibility that the mutation alters the conformational distribution of the holo form in such a way to reduce the overlap (i.e., the present analysis assumes the mutation does not impact the holo conformations). The comparisons to mutant-holo are necessary to support the authors' main conclusion.

We thank the reviewer for this observation that allows us to clarify a point that was not entirely clear in our previous submission and to add a more nuanced discussion.

Our choice to compare the mutant-apo with the holo-wt was motivated by the consideration that the mutant proteins we selected are defective in lipid transport. Hence, we were (are) worried that simulations of holo-mutant could be unphysical, as this state might not exist physiologically and not be a thermodynamic minimum. Hence, we compared the apo-mutant forms with the holo-wt as this state is our reference “ground truth” for lipid transport activity, since we know that the wild type protein is able to transport lipids.

However, as requested by this Reviewer, we have now added simulations of the mutant-holo and compared with those of mutant apo. In 3 out of 4 cases (the sole exception being GRAMD1A) the KL overlap between mutant-apo and mutant-holo is higher than that of wt-apo and mutant-apo. These results are consistent with our hypothesis and with the results we have previously shown regarding the overlap between mutant-apo and wt-holo. In addition, the only result that doesn’t fit with our hypothesis, that of GRAMD1A, can be easily explained, as both apo-mutant and holo-mutant conformational distribution deviate significantly from those of the wt, likely destablizing the “closed” state and resulting in a conformation that is not conducive to lipid transport.

We have now added the new results requested by the Reviewer in SI and added the following, more nuanced, explanation in the main text:

*“To further investigate the relation between LTP’s conformational plasticity and lipid transport, we next identified three LTD mutants of proteins in our database that have been characterized to decrease lipid transport rates experimentally (GRAMD1A 5P^47^, CPTP V158N^12^ and PITP P78L^48^). For these proteins, we performed MD simulations of the mutant apo* ***and holo forms, to investigate whether introducing mutations alters the conformational distribution of these states and to what extent it affects the overlap between apo and holo populations****. To do so, we computed the KL divergence between the apo form of these mutants and their respective wild-type holo form,* ***that we take as "ground truth" for the transport activity (Fig. 5b) as well as the KL divergence between the apo and holo mutant forms (Fig. S7).*** *These analyses confirm that for all mutants, which are defective in lipid transfer, KL divergence increases with respect to the wild-type (Fig. 5b),* ***with the sole exception of the GRAMD1A 5P when holo and apo mutants are compared (Fig. S7). However, it is likely that this apparently negative result originates from the large effect of the 5P mutation on protein conformational plasticity, as both apo-mutant and holo-mutant conformational distributions deviate significantly from those of the wild type (Fig. 5b and S7). This results in enhanced destabilization of the lipid-bound state, hence yielding a conformation that is not conducive to lipid transport. Furthermore, there is no indication that holo-mutant conformations represent bona fide thermodynamic minima, as the initial conformations for these mutants can only be generated starting from the wild type structures (see Methods), and considering that these mutants are heavily defective in lipid transport and hence might not bind and transport lipids in native conditions.”***

Fig 2 presents normalized RMSFs. Are the RMSF magnitudes similar across all proteins investigated or are there LTPs that are overall more rigid or flexible than others?

Following this reviewer’s request, we add below the RMSF values without normalization. With few exceptions (N-terminal part of Osh4, Sfh1, TTPA), all proteins have a similar range of RMSF values, with less mobile regions around 0.1-0.2 nm, and more mobile regions around 0.3-0.6 nm. Because of this, we have opted to keep the normalized RMSF in the main manuscript. As an interesting side note, Sfh1, one of the most challenging proteins in our database, is also one of the most rigid. This high rigidity of Sfh1 could explain the bad correlation between membrane binding and PC1 we observe for this specific protein. However, given the insufficient statistics (n=1), we prefer to not speculate about this point in the main text.

**Figure R1.** Non-normalized RMSF for all proteins in our database.

PC1 certainly explains the most variance, albeit typically less than 50%, and well captures differences in conformational changes that seem functionally relevant. Residue's contributions to PC1 are strikingly similar to their RMSFs (Fig 2b vs Fig 3a). Differences in PC1 do not fully explain differences in cavity volume of apo vs holo forms (Fig S6), suggesting that some relevant conformational features may not be captured by PC1 alone. For these reasons, it would be great if the authors could comment about and/or illustrate the features captured by PC2 in the SI.

We certainly agree that while PC1 explains most of the variance, additional features can be captured by including additional principal components. To this end, we analyzed other modes but, as shown in the manuscript, we found PC1 to be sufficient to provide a common and general mechanism for all protein. In most cases, the contributions to PC1 and PC2 are rather similar (see Figure R2), so, although PC2 will capture other subtle movements which are underrepresented in PC1, the information gained from including PC2 in our analysis would be relatively low. Hence, our overall conclusions would not change, but it would certainly make the results from the manuscript harder to interpret for the average reader.

**Figure R2.** Comparison of PC1 and PC2 contributions for all proteins in our database.

To clarify this point, we have added the following comment in the Conclusion section:

*“First, our atomistic sampling of the conformational dynamics of LTDs is finite, and even though PCA analysis allows to describe the essential dynamics of the LTDs, we can’t completely describe larger conformational changes that might occur on longer timescales, such as, for example the opening/closing of the lid occurring in Osh proteins upon membrane binding^11, 14, 22^.* ***In addition, while the first principal component (PC1) explains a high percentage of the conformational variance, other, more subtle, conformational features might not be captured by PC1 alone and would require contribution from other principal components.”***

I appreciate the authors edits to clarify their focus on conformational plasticity, a thermodynamic property. Nevertheless, on p. 8, PCA is described as filtering out "high-frequency fluctuations occurring during the protein dynamics" to "identify… slower motions," and "explaining most of the variance in the dynamics." While PCA is evocative of this (and often described with similar phrasing in the literature), it does not explicitly account for temporal correlations and dynamics, such as done in tICA. To avoid misleading readers (especially given the increasingly imprecise and ambiguous use of the terms "conformational ensemble" and "dynamics" in the current biomolecular modeling literature), I suggest the authors revise these statements to emphasize PCA's utility for capturing dominant, large-scale variations in protein conformations sampled during the simulations.

We have added the following, more neutral, description in the text:

*“To this end, we analyzed the MD simulations using principal component analysis (PCA) (Fig. 3),* ***as this technique can be used to capture dominant, large-scale variations in protein conformations sampled during the MD simulations^41, 42^.”***

It would aid readers if the authors specifically state in the abstract that the "common feature" is that LTPs interconvert between different conformational states (or a similar phrasing equating this feature to their conformational plasticity).

We have now modified the abstract according to this suggestion.

Please increase the size of all axes' labels and tick marks in Fig 2c, 3b, bar plots of KL divergence shown in Fig 5 and 6b, and Fig S1.

We modified the figures accordingly.

On p. 3, I suggest the wording "transferring a lipid between two membranes" would be preferable to "across two membranes" to avoid any confusion with lipid flip-flop.

We modified the text accordingly.

On p. 10, I suggest the wording "regions… are involved in large collective motions" over "display" to emphasize membrane binding regions' contributions to conformational changes extending over the entire protein, not just membrane-bound regions, as highlighted by the authors in their response.

We modified the text accordingly.

A few minor grammatical issues need addressing in (i) the abstract: "allowing to interpret and design experimental strategies"; (ii) p. 3: "allowed to fine-tune the mechanism"; (iii) p. 4: "implications with" should be "implications for"; and (iv) p. 13: "on LTD conformational landscape" should be "on a LTD's…".

We modified the text accordingly.
